# Mechanistic analysis of an asymmetric palladium-catalyzed conjugate addition of arylboronic acids to β-substituted cyclic enones†

**DOI:** 10.1039/c4sc03337j

**Published:** 2014-12-17

**Authors:** Cornelia L. Boeser, Jeffrey C. Holder, Buck L. H. Taylor, K. N. Houk, Brian M. Stoltz, Richard N. Zare

**Affiliations:** a Department of Chemistry, Stanford University Stanford CA 94305 USA rnz@stanford.edu; b The Warren and Katharine Schlinger Laboratory for Chemistry and Chemical Engineering, Division of Chemistry and Chemical Engineering, California Institute of Technology Pasadena California 91125 USA; c Department of Chemistry and Biochemistry, University of California Los Angeles California 90095 USA

## Abstract

An asymmetric palladium-catalyzed conjugate addition reaction of arylboronic acids to enone substrates was investigated mechanistically. Desorption electrospray ionization coupled to mass spectrometry was used to identify intermediates of the catalytic cycle and delineate differences in substrate reactivity. Our findings provide evidence for the catalytic cycle proceeding through formation of an arylpalladium(ii) cation, subsequent formation of an arylpalladium–enone complex, and, ultimately, formation of the new C–C bond. Reaction monitoring in both positive and negative ion modes revealed that 4-iodophenylboronic acid formed a relatively stable trimeric species under the reaction conditions.

## Abstract 2

Mechanistic insight into a Pd-catalyzed conjugate addition reaction was gained using desorption electrospray ionization coupled to mass spectrometry.

## Graphical abstract

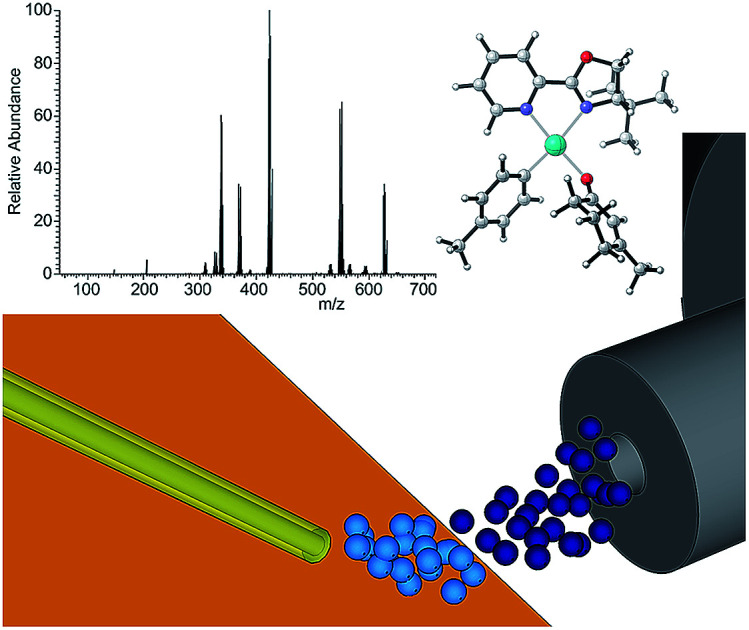

## Introduction

**Scheme 1:**
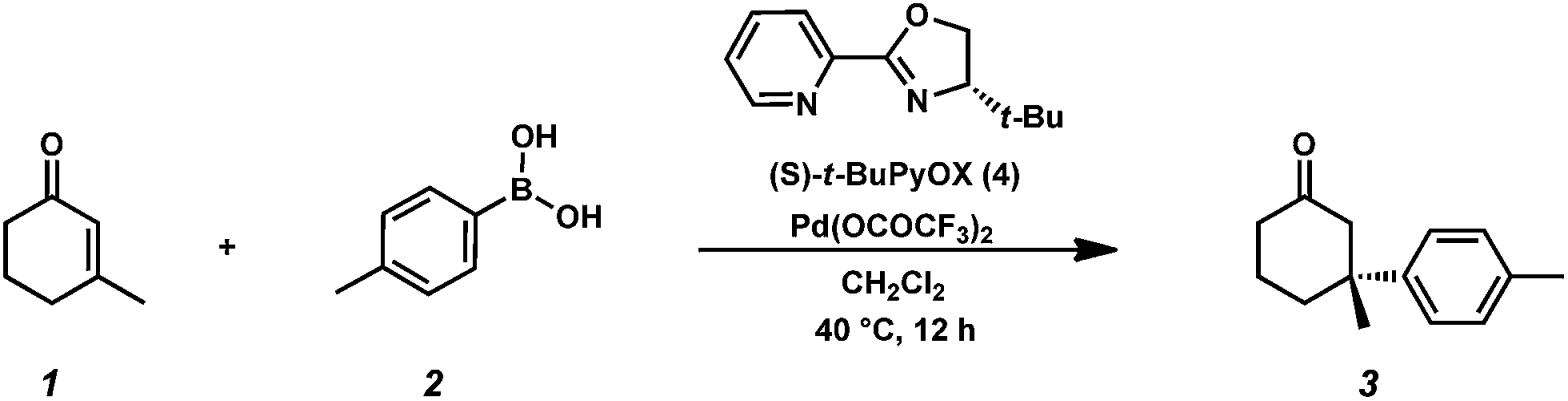
Asymmetric conjugate addition of arylboronic acids to cyclic enones catalyzed by the combination of Pd(OCOCF_3_)_2_ and *t*-BuPyOx.

Asymmetric conjugate addition has been used to develop enantioselective pathways for the construction of all-carbon quaternary centers.^1–5^ Reaction development remains a challenging area of research because many established conjugate addition reactions rely on high-cost catalysts and require anhydrous conditions. Palladium-catalyzed conjugate addition reactions are an ongoing field of research.^6–8^ The Stoltz group recently developed a palladium-catalyzed asymmetric conjugate addition reaction of phenylboronic acids and cyclic enones.^9–11^ In contrast to alternative methods,^12–17^ this reaction is tolerant to oxygen and moisture. All reagents and catalyst components are bench-stable, and the reaction is conducted under an air atmosphere with 5 equiv. H_2_O added to facilitate catalyst turnover. Using the chiral pyridinooxazoline (PyOx) ligand (*S*)-*t*-BuPyOX (4) produces enantioenriched product in up to 99% yield (Scheme 1). The synthesis of *t*-BuPyOX can be achieved using a simple, two-step procedure.^18^

In desorption electrospray ionization (DESI)^19^ coupled to mass spectrometry (MS) a reaction can be initiated by spraying charged droplets of a reagent onto a substrate that has been deposited on a surface.^20–25^ Turnover occurs inside secondary microdroplets that are scattered from the sample surface. These secondary droplets are directed toward the inlet of a mass analyzer. As the reaction is quenched by evaporation of the droplets, gas-phase ions are formed and mass analyzed. Generally, reaction times on the order of milliseconds^26,27^ can be accessed using this technique. However, DESI is not limited to short reaction times. Timescales on the order of minutes or hours can be assessed by sampling a reaction flask, spotting the aliquot on the surface and spraying it with pure solvent. This method has significant advantages as a sampling method over conventional direct infusion electrospray ionization. By taking a spectrum of the blank surface before analyzing the sample in the same run, potential background ions can easily be subtracted. Additionally, contamination of the syringe, tubing and fittings used for sample transfer is minimized. Coupling DESI (experimental setup is shown in Fig. S1†) to a high-resolution Orbitrap mass spectrometer allows for reliable identification of the signals by their *m*/*z* ratios and isotopic profiles.

The Stoltz group, in collaboration with the Houk group, recently reported computational and experimental evidence for a mechanistic pathway involving the formation of a cationic arylpalladium(ii) species, and has identified this organometallic complex as the species involved in the turnover-limiting and enantiodetermining carbopalladation of the enone to form the new C–C bond.^10^ Furthermore, the lack of a nonlinear effect excluded the participation of (ML)_2_ dimer complexes in the catalytic cycle and implicated a monomeric palladium–ligand complex as the active catalyst.^28–30^ Herein we report the use of DESI coupled to high-resolution MS to investigate the mechanism of the reaction. We have identified intermediates that strongly support the proposed mechanistic pathway and thereby substantiate the significance of DESI as an analytical tool in reaction development and analysis.

## Results and discussion

### Mechanistic analysis

**Fig. 1:**
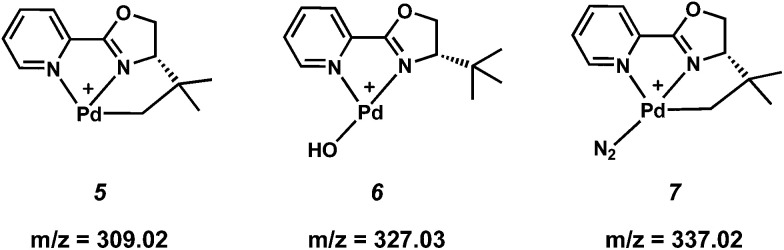
Suggested structures for selected species identified when a solution of ligand 4 and Pd(OCOCF_3_)_2_ is analyzed by DESI-MS.

The reaction occurs under mild conditions at 40 °C using either 1,2-dichloroethane or dichloromethane as the solvent. The catalyst is formed *in situ* by stirring Pd(OCOCF_3_)_2_ with ligand 4. In previous reports, it was disclosed that the addition of salts that contain a weakly coordinating anion, such as NH_4_PF_6_, and the addition of H_2_O as the putative protonating agent increased the rate of reaction.^9,10^ Unless otherwise noted, samples were prepared in 1,2-dichloroethane. However, due to toxicity and spray instability of the latter, dichloromethane was used as spray solvent in all DESI experiments. Initial investigations focused on formation of the catalyst. Pd(OCOCF_3_)_2_ was mixed with PyOx (4) in 1,2-dichloroethane (both 0.5 mmol) and spotted on paper. We also used PTFE and glass in place of paper but observed no differences. Data were acquired in positive ion mode. Spraying dichloromethane onto Pd-source and ligand gave the mass spectrum shown in Fig. S2.† We identified ligand 4 as both H^+^ (*m*/*z* 205.13) and Na^+^ (*m*/*z* 227.11) adducts. The catalyst appears as singly charged ion 5 at *m*/*z* 309.02 (Fig. 1).

We attribute the loss of a hydrogen atom to C–H palladation of the *t*-butyl group of the ligand.^31^ We also identified the hydroxide-bound Pd-cation [(PyOx)Pd(OH)]^+^ (6) at *m*/*z* 327.03. The C–H palladation product 5 seems to easily coordinate N_2_ to form complex 7, detected at *m*/*z* 337.02. This capture process happens either in solution, or as a result of the ionization process. It could also be speculated that N_2_ is picked up in the flight path from inlet to detector, as ions pass a curved linear trap filled with nitrogen before being injected into the Orbitrap mass analyzer. Interestingly, isolating complex 5 in the ion trap (with an isolation window of 1 mass unit) leads to concurrent isolation of complexes 6 and 7 (Fig. S3†). Accordingly, isolation of hydroxide 6 leads to concurrent isolation of nitrogen-bound species 7. We speculate that complexes 6 and 7 could be artifacts that are formed by the palladium–ligand complex in the spectrometer due to vacant coordination sites on the palladium. Formation of complexes 6 and 7 appears to depend on the voltage applied to the syringe during DESI experiments (Table S1†). Decreasing the voltage leads to an overall decrease in the signal, while the ratio of complexes 6 and 7 compared to C–H palladation adduct 5 increases significantly. This finding suggests that experiments should be carried out at 5 kV in order to optimize the signal of 5. Other major signals correspond to [(PyOx)Pd(OCOCF_3_)]^+^ (8) at *m*/*z* 423.01, [(PyOx)_2_PdCl]^+^ at *m*/*z* 549.12 and [(PyOx)_2_Pd(OCOCF_3_)]^+^ at *m*/*z* 627.14. Species incorporating two ligands are only detected when Pd and ligand are premixed, followed by mass analysis. If the Pd source is spotted on the surface and sprayed with a ligand solution, no ions involving two ligands could be identified. This result suggests that they are formed at increased incubation times.

**Scheme 2:**
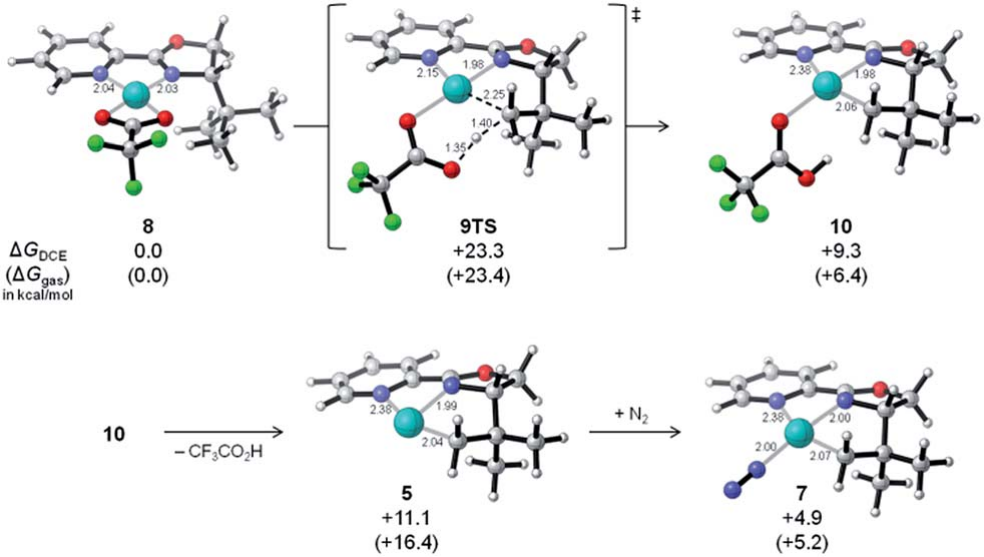
Computed mechanism for formation of C–H palladation products 5 and 7.

In order to investigate the feasibility of the C–H palladation of the *t*-butyl group in complex 5 we performed density functional theory calculations. Here we report Gibbs free energies calculated in the gas-phase using the M06 density functional. Solvent effects were also calculated using the SMD solvation model for 1,2-dichloroethane (dichloromethane gives nearly identical results), since it is not known which steps take place in solution or in the flight path to the mass spectrometer. Our calculations began with the cationic trifluoroacetate complex [(PyOx)Pd(OCOCF_3_)]^+^ (8, Scheme 2). This complex was observed in the DESI spectrum of the previous experiment (*m*/*z* 423.01, Fig. S2†). C–H palladation proceeds *via* a concerted metallation–deprotonation mechanism through 9TS. The barrier for this reaction (23 kcal mol^−1^) is reasonable for a room-temperature process. The C–H palladation initially forms complex 10, from which trifluoroacetic acid can dissociate to form the observed complex 5. In this complex the ligand is tridentate, but distorted significantly out of planarity. In addition, the Pd–N_pyridine_ bond is elongated by about 0.3 Å (relative to complex 8) to accommodate the newly formed Pd–C bond. Nevertheless, a conformer of 5 lacking the Pd–N_pyridine_ bond was calculated to be 5 kcal mol^−1^ higher in energy than complex 5 (Scheme S1†). The vacant coordination site makes complex 5 a relatively high-energy intermediate, particularly in the gas phase, making coordination of N_2_ to form complex 7 very favorable. Notably, all C–H palladated intermediates examined are higher in energy than the precursor complex 8, suggesting that the C–H activation occurs as a result of the ionization process. These data strongly suggest that metallacycles 5 and 10 do not participate in the catalytic cycle and are also not likely pathways of catalyst decomposition under the catalytic reaction conditions.

We also considered C–H palladation occurring from a neutral complex (PyOx)Pd(OCOCF_3_)_2_; however, this pathway is calculated to be much higher in energy (Scheme S2†). Calculations for complex 6 are shown in Scheme S3.†

**Fig. 2:**
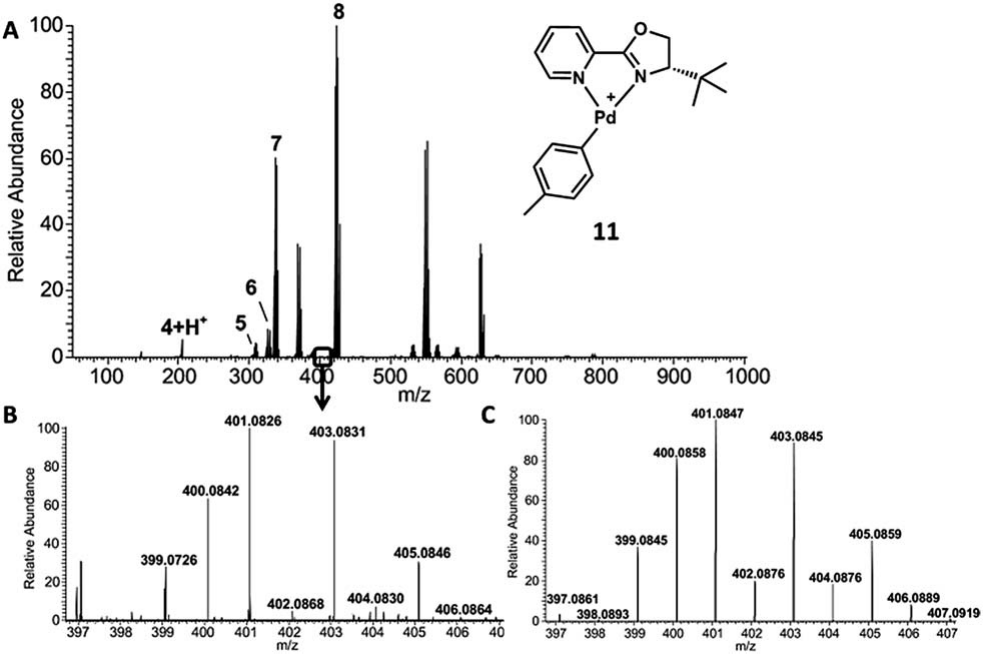
(A) 1 mmol Pd source and ligand sprayed with 0.1 mmol *p*-tolylboronic acid. (B) Experimental spectrum of 11. (C) Simulated spectrum of 11.

We continued our DESI-MS studies by spraying *p*-methylphenylboronic acid (2, 0.1 mmol) onto the pregenerated catalyst. In addition to complexes 5, 6, 7 and 8, we found evidence of the arylpalladium cation 11 (Fig. 2), which results from the putative transmetallation of arylboronic acid 2 with the cationic Pd(ii) species in the reaction mixture.

When catalyst and arylboronic acid 2 are premixed for 5 min and sprayed with pure dichloromethane, the signal of the arylpalladium intermediate 11 (*m*/*z* 401.08) increases significantly. Additionally, a signal at *m*/*z* 605.21 was detected, which we attribute to [(PyOx)_2_Pd(*p*-tol)]^+^ (*m*/*z* 605.21), an arylpalladium-cation bearing two ligands. Surprisingly, collision induced dissociation (CID) of arylpalladium 11 leads to loss of the aryl group and formation of complexes 6 and 7 (Fig. S4†). The formation of the arylpalladium intermediate seems not to be correlated with the type of arylboronic acid used. Both electron-rich and electron-deficient arylboronic acids form the corresponding cationic arylpalladium species (Fig. S5†). We even observed arylpalladium cation formation when the catalyst solution was premixed with arylboronic acids that do not afford conjugate addition product under the typical reaction conditions (*e.g.*, 4-iodophenylboronic acid). This observation is consistent with the calculated reaction coordinate, on which transmetallation of arylboronic acid to form arylpalladium cation 11 is not the turnover-limiting step of the catalytic reaction.

**Fig. 3:**
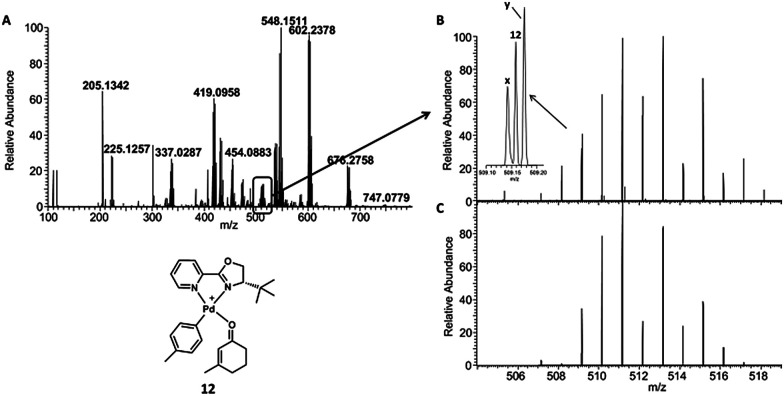
(A) Incubated reaction sampled and mass analyzed after 8 h. (B) Experimental spectrum of 12. The signal is overlayed by x [(PyOx)_2_Pd)]^+^ (*m*/*z* 513.14)‡‡This complex undergoes C–H insertion of palladium into one of the ligands. and y [(PyOx)Pd(phenyl)_2_]NH_4_^+^ (*m*/*z* 510.17). (C) Simulated spectrum of 12.

Because neither spraying enone 1 onto a mixture of pre-generated catalyst and arylboronic acid 2 nor spraying a mixture of enone 1 and arylboronic acid 2 onto the catalyst indicated any reaction progress, we decided to further investigate incubated reaction mixtures in order to access longer reaction times. After a reaction time of 8 h, a 100-fold diluted aliquot was spotted on the surface and mass analyzed. The resulting spectrum is shown in Fig. 3A. Ketone product 3 was identified as Na^+^-adduct (*m*/*z* 225.12). The arylpalladium cation 11 is also present. The signal around *m*/*z* 512 reveals three major species, one of them being the proposed arylpalladium–enone complex 12 at *m*/*z* 511.15 (Fig. 3B and C).

However, a product-enolate–palladium complex would also correspond to *m*/*z* 511.15 (Fig. S6†). To distinguish 12 from this alternative compound, we carried out CID experiments. Fragmentation of 511.15 leads to loss of CO, possibly expelled from the enone (Fig. S7†). Further, the compound undergoes neutral loss of the methylphenyl group which leads to formation of the enone–palladium complex (*m*/*z* 419.09). It is unlikely that the loss of a methylphenyl group could occur from the product-enolate–palladium complex because this would require carbon–carbon bond cleavage to occur. However, loss of the methylphenyl group from arylpalladium complex 12 is much more likely because this only requires the cleavage of the longer and weaker carbon–palladium bond.^32^ Considering the relatively low CID collision energy of 10% (manufacturers unit), we consider complex 12 as the more likely structure. In addition, our calculations indicate that the enone complex 12 is more stable than the product–enolate complex (Scheme S4†). The putative enolate structure is unlikely to be observed by MS because it is formed after the rate-limiting step of the calculated reaction coordinate.^10^ Conversely, proposed enone complex 12 precedes the rate-limiting step, and may even be the resting state. Furthermore, if *m*/*z* 511.15 were an enolate complex, then CID experiments would easily cleave the palladium–oxygen bond and lead to the observation of a peak corresponding to the *m*/*z* of the product ketone or free enolate. These are not observed. However, a mass corresponding to the aryl fragment and enone–palladium complex are observed. These data strongly support the reported structure of complex 12 over the alternative palladium–enolate complex.

Several arylboronic acids were tested for formation of intermediates and product. Table 1 shows the intermediates that were generally observed for a variety of arylboronic acid substrates. No 4-chlorophenylpalladium–enone-complex could be identified, potentially caused by the lower stability of the complex compared to other arylboronic acids. However, product formation was observed for this reaction. It is intriguing that no product formation was observed for 4-iodophenylboronic acid. Mechanisms for reaction inhibition are not known. Using the alternative substrate 4-hydroxy-chromone instead of 3-methylcyclohexenone (1) led to similar results including the inability to form product using 4-iodophenylboronic acid (Table S2†). No dimeric palladium-species were detected. This corroborates previous studies indicating that the active catalyst is most likely a monomeric Pd-complex.

**Table 1:** Observed intermediates and product formation for the addition of arylboronic acids to 1 at a reaction time of 10 h. Relative intensities are in parentheses

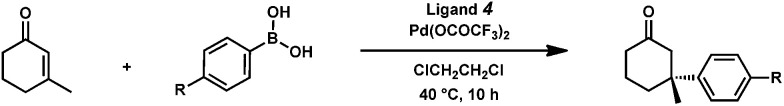				
Entry	*R* =	*m*/*z*_arylpalladium–cation_	*m*/*z*_arylpalladium–enone_	*m*/*z*_product_^a^
1	4-Me–	401.08 (6.8)	511.15 (8.7)	225.12 (8.5)
2	4-Cl–	421.03 (0.9)	(0)	245.07 (2.8)
3	4-BnO–	493.11 (4.7)	603.18 (1.2)	317.15 (20.8)
4	4-I–	512.96 (1.5)	(0)	(0)

a Observed as Na^+^-adducts.

### Reaction monitoring

**Fig. 4:**
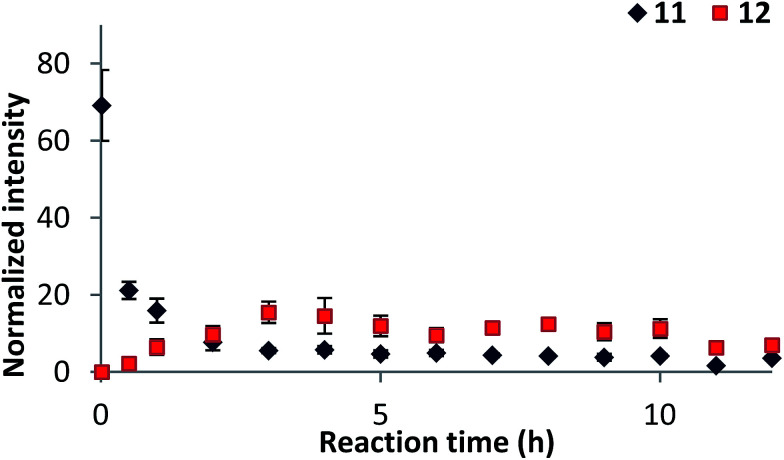
Monitoring of intermediates 11 and 12 by DESI-MS.

The identification of intermediates along with the inability to form product using 4-iodophenylboronic acid triggered us to look at reaction progress over time. Initial experiments focused on monitoring the conjugate addition reaction of *p*-methylphenylboronic acid (2) with 3-methylcyclohexenone (1) over the time course of 12 h. Positive ion mode was used to monitor product formation and intensities of intermediates. Negative ion mode was used to monitor the speciation of arlyboronic acid 2. The LTQ-XL Orbitrap does not allow for fast polarity switching. Therefore a separate reaction was carried out for each polarity on different days, but otherwise similar conditions. The increase of product *versus* decrease of substrate is shown in Fig. S8.†Fig. 4 shows the distribution of the two key intermediates, arylpalladium cation 11 and enone-complex 12. Interestingly, while arylpalladium cation 11 constantly decreases over the course of the reaction, enone-complex 12 increases up to 3 h, then plateaus and finally decreases after 11 h. Pd(OCOCF_3_)_2_, boronic acid 2 and ligand 4 are preincubated for 2 min before addition of enone 1, which marks the starting point of the reaction. This accounts for the high initial intensity of arylpalladium cation 11. Upon addition of enone 1 to the mixture, arylpalladium cation 11 reacts with substrate 1 to form enone-complex 12. When the reaction approaches completion around 10 h, the concentration of enone-complex 12 decreases as well.

**Fig. 5:**
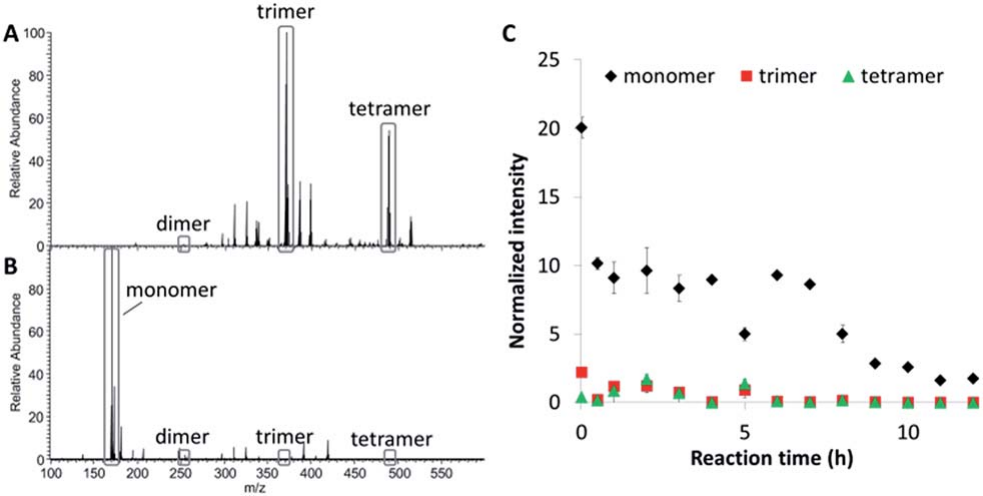
(A): 1 mmol 2 spotted on a surface and sprayed with CH_2_Cl_2_. Dimer, trimer, and tetramer were detected as OH-adducts in negative ion mode. (B) Distribution of 2 after reaction completion. (C) Reaction monitored over 12 h. Ion intensities are normalized to the total ion count.

The negative ion trace of the reaction was monitored under slightly modified conditions. Addition of NH_4_PF_6_ to the reaction mixture caused suppression of signals corresponding to arylboronic acids caused by the large signal at *m*/*z* 144.96 corresponding to PF_6_^−^. Consequently, no NH_4_PF_6_ was added for this experiment. Preliminary experiments involving a freshly made solution of arylboronic acid 2 in 1,2-dichloroethane showed an interesting distribution between monomer and dehydrated dimeric, trimeric and even tetrameric forms of 2. Under the reaction conditions, the trimeric species is dominant, followed by the tetramer (Fig. 5A). The dimer signal is hardly visible, whereas no signal corresponding to monomeric 2 can be identified. Similar behavior was observed in solutions of other types of arylboronic acids, including 4-iodophenylboronic acid (Table S3†). In solution, arylboronic acids tend to undergo dehydration leading to formation of cyclic trimers.^33^ The signal corresponding to a dehydrated tetramer, however, was surprising. At this point it is not clear whether its structure is cyclic or acyclic. We attribute its formation to the ionization process. During this process, solvent continuously evaporates which likely promotes dehydration compared to solution phase. Following dehydration, the tetramer could be generated by concentration effects, a common phenomenon in DESI-MS that leads to detection of analyte clusters. Interestingly, when arylboronic acid 2 is dissolved in dichloromethane instead of 1,2-dichloroethane, the monomeric signal is instead the base peak in the spectrum (Fig. S9†). Previous experiments, however, proved 1,2-dichloroethane to be superior to dichloromethane with regard to yield and reaction time.^10^

During the reaction, however, the distribution shifts toward the monomeric state. In fact, upon start of the reaction, the signals corresponding to trimer and tetramer readily decrease after only a few min of reaction time (Fig. 5C). At the end of the reaction (Fig. 5B), the monomer is the most abundant ion, leaving trimer and tetramer almost fully consumed. We think that trimerization of arylboronic acid 2 is reduced by premixing it with Pd(OCOCF_3_)_2_ and PyOx ligand 4. In solution, arylpalladium cation 11 is readily formed, which coordinates enone substrate 1 in the next step of the proposed reaction cycle. Trimeric and tetrameric forms of arylboronic acid 2 are assumed to convert to the monomeric form, which subsequently undergoes transmetallation to form the arylpalladium cation 11. This hypothesis explains the rapid decrease of trimer and tetramer. Other arylboronic acids gave the same distribution before and after the experiment (Table S3†). Interestingly, 4-iodophenylboronic acid also shows a signal corresponding to its monomer after the reaction. However, the significantly higher ion intensities are observed for its trimeric and tetrameric form both, before and after the reaction. 4-iodophenylboronic acid has been shown to be nonreactive for this type of transformation in earlier experiments; no product formation was observed after 12 h reaction with either enone 1 or 4-hydoxy-chromone under standard reaction conditions. While we cannot fully explain the observed lack of reactivity, we posit that the iodophenylboronic acid trimers, the large quantity of side products formed, and the lack of conjugate addition reaction may indicate a possible change in the turnover-limiting step of the catalytic cycle when 4-iodophenylboronic acid is the substrate.§§Arylpalladium cation 11 is detected when 4-iodophenylboronic acid is used as the substrate, albeit in low quantities (see Fig. S5†). Protodeboronation to form iodobenzene is the major product of the reaction, and biaryl homocoupling is observed as a minor product. After extended reaction times trace conjugate addition product can be observed. From these data we conclude that transmetallation occurs when the catalyst is treated with 4-iodophenylboronic acid, but undesired pathways outcompete the conjugate addition reaction. This observation may indicate a possible change in turnover-limiting step – or possible change in the resting state – of the catalytic cycle.

## Conclusions

We monitored the Pd-catalyzed asymmetric conjugate addition reaction of arylboronic acids to cyclic enones by means of DESI-MS. This powerful technique revealed the formation of two important intermediates, corroborating the reaction mechanism previously proposed by the Stoltz and Houk groups. Our experiments delineated differences in reactivity of arylboronic acid substrates, which might be related to stability of the unreactive arylboronic acids in trimeric and potentially tetrameric forms. Our findings will facilitate future reaction optimization and underline the importance of using DESI-MS in reaction analysis and development.

## Experimental section

Pd(OCOCF_3_)_2_, 3-methylcyclohexen-2-one, 4-hydroxy-chromone and all arylboronic acids was purchased at Sigma-Aldrich. The ligand (*S*)-*t*-BuPyOX was synthesized according to reported literature procedures.^18,34^ Unless otherwise noted, all reactions were carried out in dichloroethane. The reaction conditions for reaction monitoring and Table 1 and S2† were as follows: Pd(OCOCF_3_)_2_ (1 mg, 0.003 mmol), (*S*)-*t*-BuPyOX (0.6 mg, 0.003 mmol), NH_4_PF_6_ (3.1 mg, 0.019 mmol) and arylboronic acid (0.125 mmol) were dissolved in 250 μL dichloroethane and stirred for 2 min at room temperature. Conjugate acceptor substrate (0.06 mmol), H_2_O (10 μL) and an additional 250 μL dichloroethane were added. The mixture was stirred in an oil bath at 40 °C for the respective amount of time. Before analysis, the mixture was diluted 100 times. An LTQ-XL Orbitrap mass spectrometer (Thermo Fisher Scientific) was coupled to the homebuilt DESI-source (described in Fig. S1†). Unless otherwise noted, the instrument parameters were set as: resolution: 60 000 at *m*/*z* 400, 5 kV applied to syringe, temperature of inlet capillary: 250 °C. Theoretical calculations were performed with Gaussian 09 (ref. 35). Geometries were optimized with the M06-L^36^ functional in the gas phase, using an ultrafine integration grid. A mixed basis set of SDD (with ECP) for Pd and 6-31+G(d,p) for all other atoms was used. Thermal corrections were calculated from unscaled vibrational frequencies at the same level of theory for a standard state of 1 atm and 298 K. Electronic energies were obtained from single point energy calculations performed with the M06^37,38^ functional and a mixed basis set of SDD for Pd and 6-311++G(d,p) for all other atoms. Single-point energy calculations were performed both in the gas-phase and using the SMD^39^ solvation model for 1,2-dichloroethane. The 3D structures of molecules were generated using CYLView.^40^

## Supplementary Material

SC-006-C4SC03337J-s001
