# Supplementary material for: Mechanistic analysis of an asymmetric palladium-catalyzed conjugate addition of arylboronic acids to β-substituted cyclic enones
Source: Chem Sci. 2014 Dec 17;6(3):1917–22. doi: 10.1039/c4sc03337j (PMC4338963; doi:10.1039/c4sc03337j)
Supplement: SC-006-C4SC03337J-s001 [file SC-006-C4SC03337J-s001.pdf]

Electronic Supplementary Material (ESI) for Chemical Science.
This journal is © The Royal Society of Chemistry 2014

Mechanistic analysis of an asymmetric palladium-catalyzed conjugate addition of arylboronic acids to ß-substituted cyclic enones

Cornelia L. Boeser, Jeffrey C. Holder, Buck L. H. Taylor, K. N. Houk, Brian M. Stoltz, Richard N. Zare

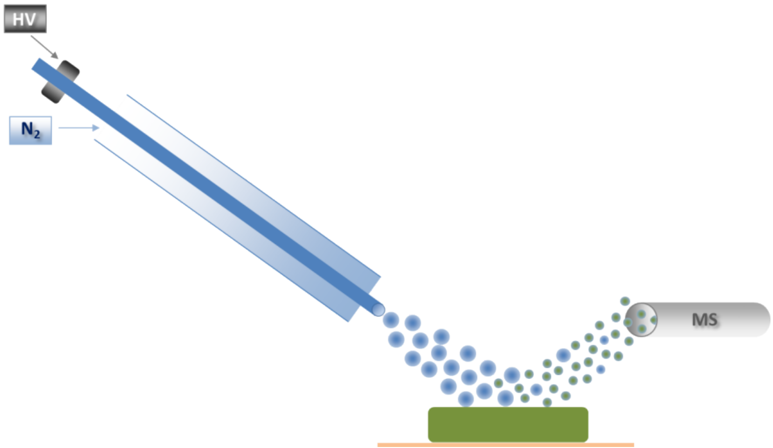

S1: DESI source: A fused silica tubing (100 µm ID, 360 µm OD) was connected to the reagent syringe using standard fingertight fittings. For spray formation, an outer metal capillary was connected to the gas supply using Swagelock T-fittings. The inner capillary extended appr. 1 mm from the outer capillary. The source was placed approximately 2 mm in front of the MS inlet at an angle of 55 degrees.

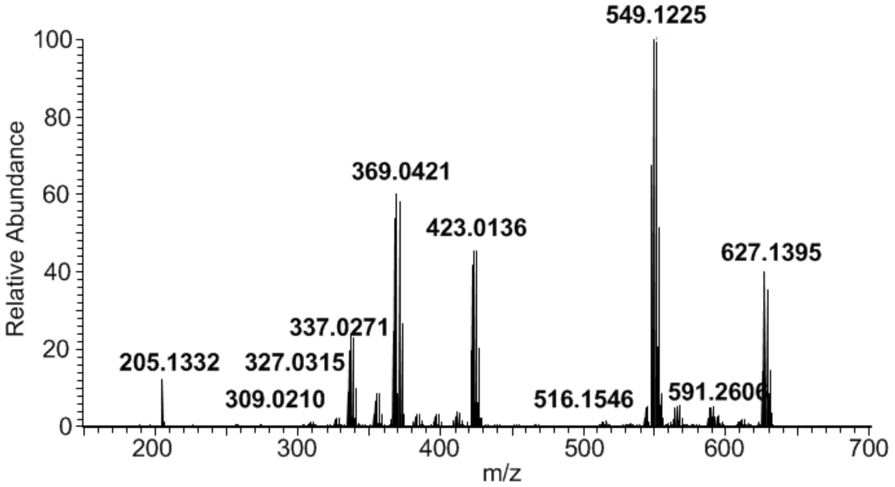

S2: DESI spectrum of 0.5 mmol $Pd(OCOCF_3)_2$ and (*S*)-*t*-BuPyOX spotted on paper and sprayed with CH_2Cl_2.

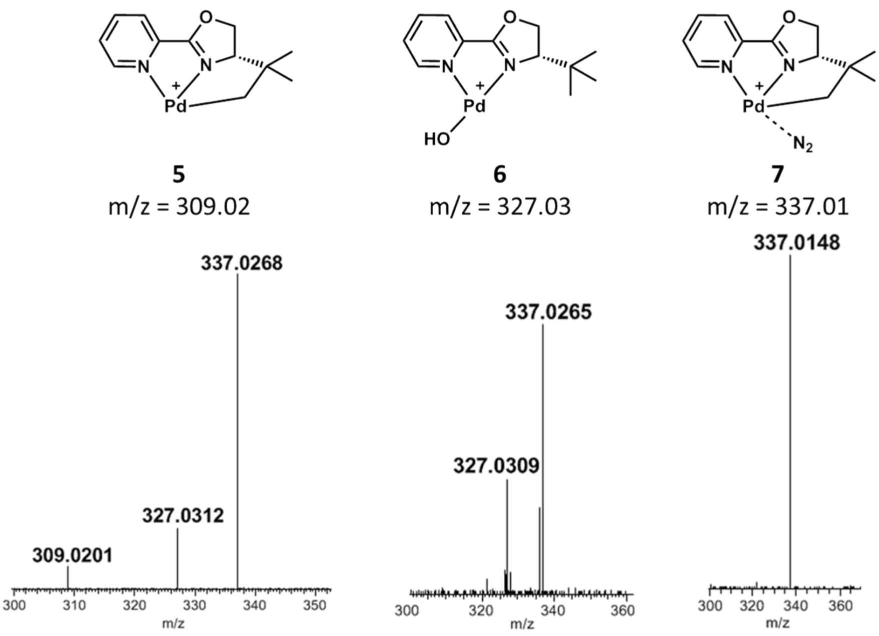

S3: Isolation patterns for species observed in sample of $Pd(OCOCF_3)_2$ and (*S*)-*t*-BuPyOX.

Table S1: Intensities of complexes **5**, **6** and **7** at varying potentials applied to the syringe carrying the spray.

Voltage	Normalized Intensities				
	5	6	7	Ratio (6/5) in %	Ratio (7/5) in %
**5 kV**	4.4	4.0	17.0	91.5	383.7
**2.5 kV**	4.0	6.1	30.2	154.3	763.9
**0 kV**	2.3	5.8	22.1	256.2	969.6

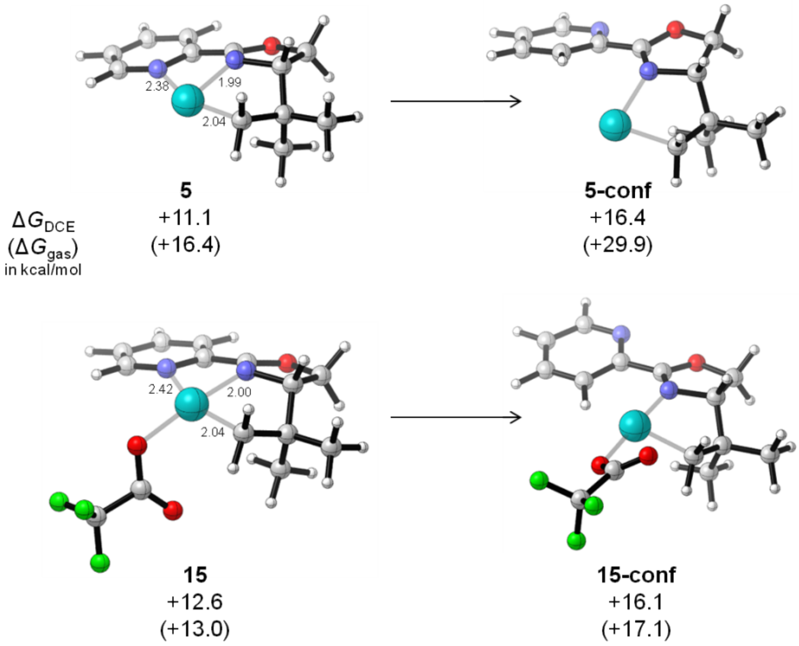

Scheme S1: Higher energy conformers of **5** and **15** lacking the Pd–$N_{pyridine}$ bond.

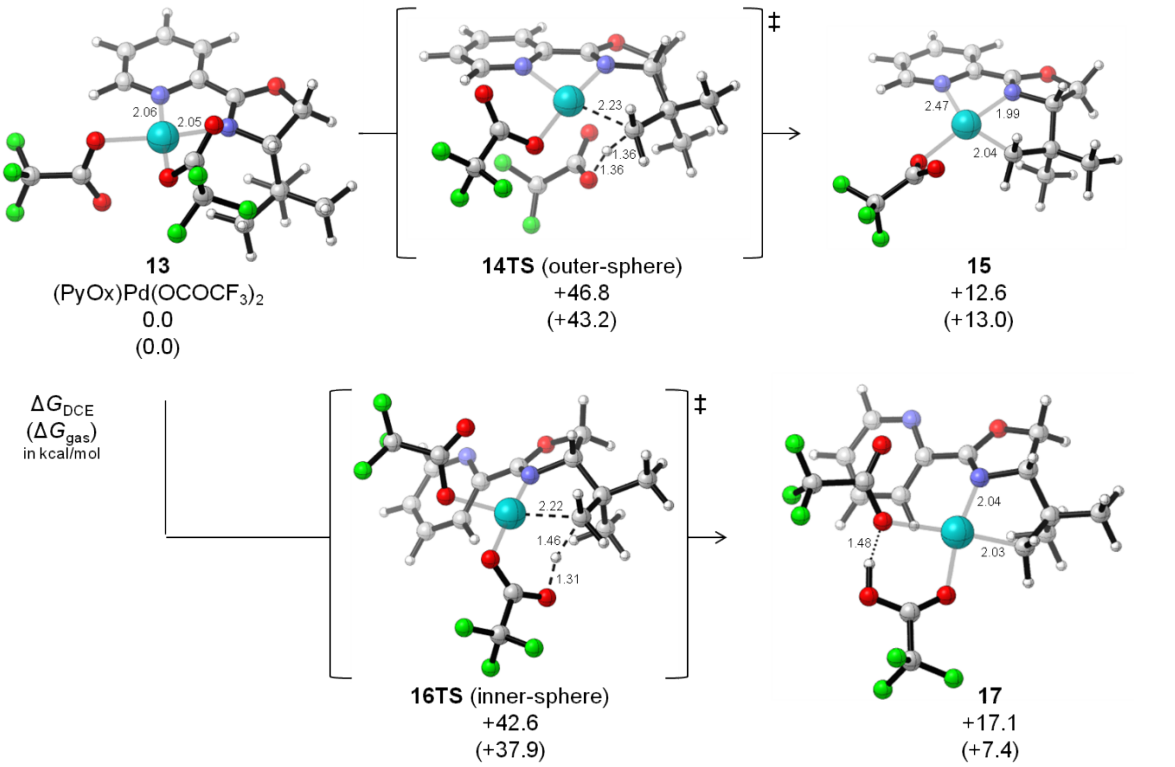

Scheme S2: Mechanism for C–H palladation of neutral complex (PyOx)Pd(OCOCF$_3$)$_2$ (**13**) proceeding through outer-sphere (**14TS**) or inner-sphere (**16TS**) mechanisms, both of which are much higher in energy than the cationic mechanism. Note: neutral complex **13** is 22.7 kcal/mol more stable than cationic complex **5** (in DCE).

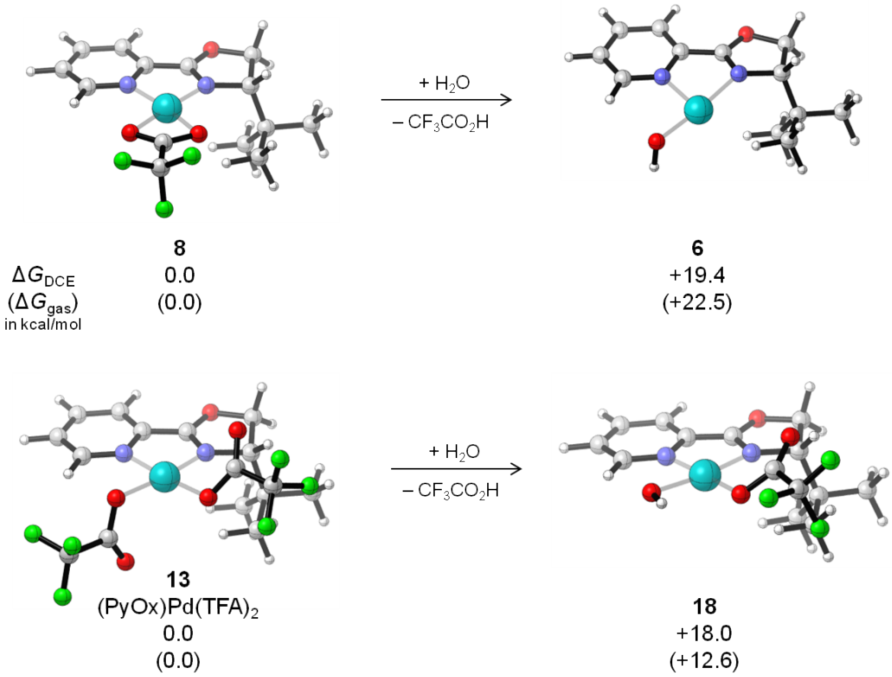

Scheme S3: Formation of hydroxide complexes cationic **6** and neutral **18**.

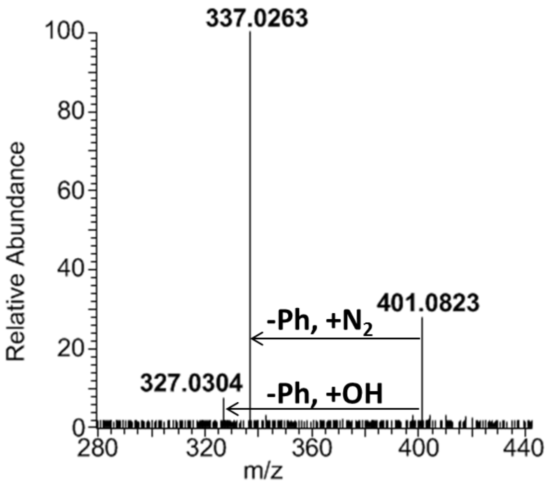

S4: CID of the arylpalladium cation **11**.

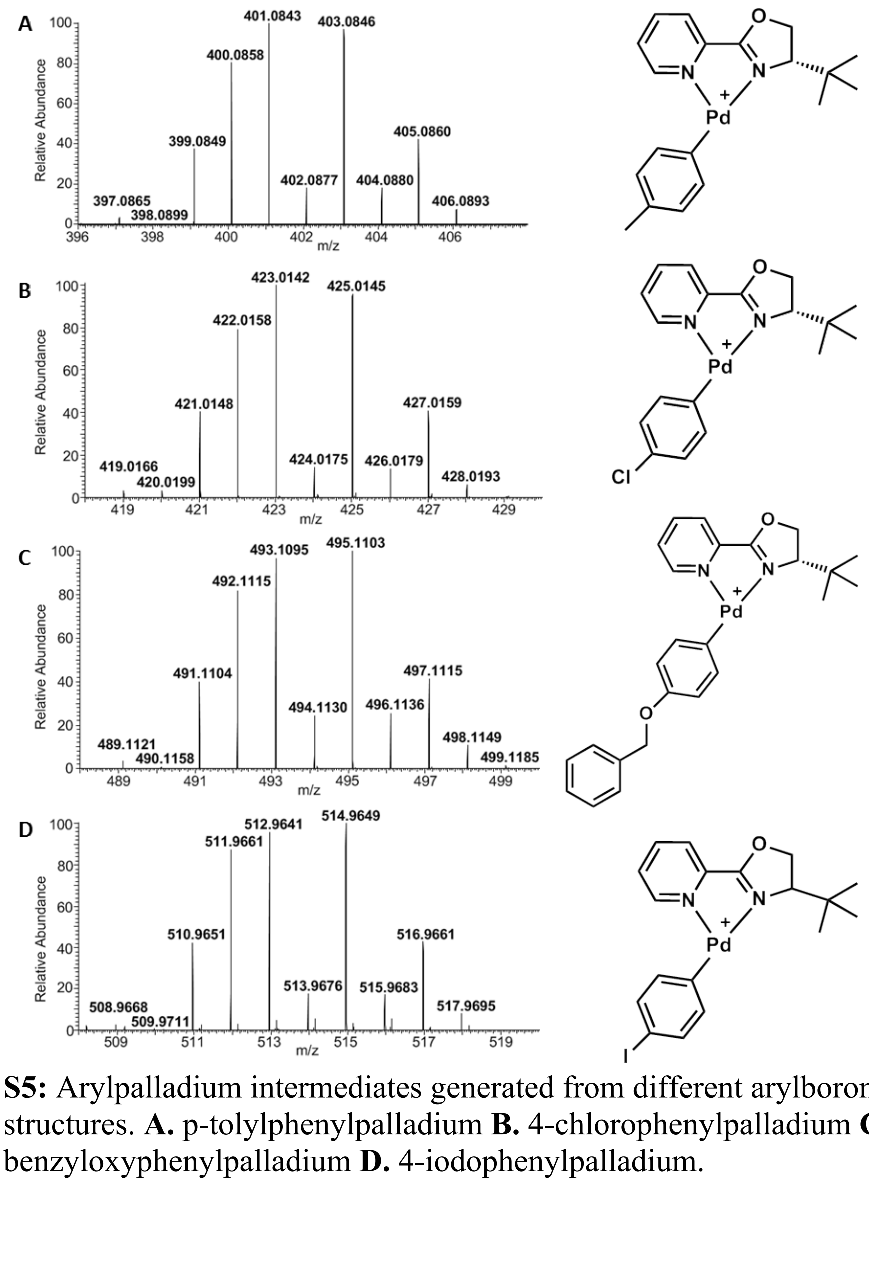

S5: Arylpalladium intermediates generated from different arylboronic acids and their putative structures. **A.** p-tolylphenylpalladium **B.** 4-chlorophenylpalladium **C.** 4-benzyloxyphenylpalladium **D.** 4-iodophenylpalladium.

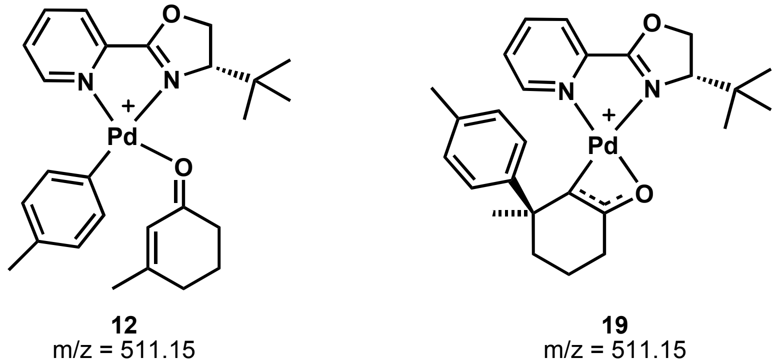

S6: Putative structures of the palladium-enone-complex **12** and the enolate-complex.

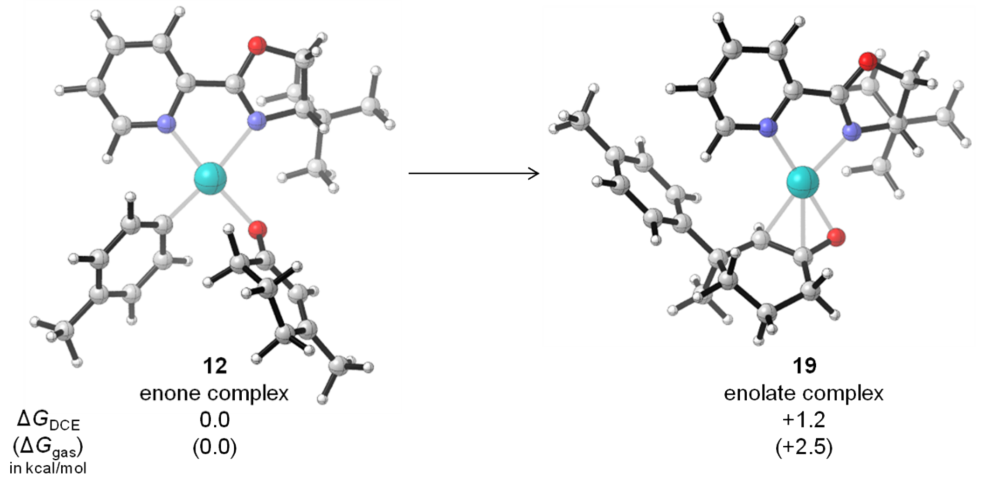

Scheme S4: Calculated structures and relative energies of palladium enone-complex **12** and alternative palladium enolate-complex **19**.

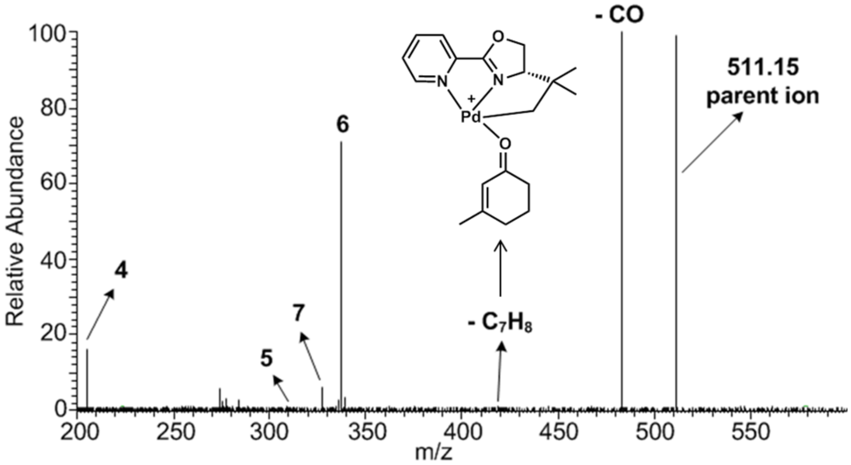

S7: CID spectrum of the arylpalladium-enone complex (top) and the enone-palladium complex (bottom), both at 10% relative collision energy.

Table S2: Observed intermediates and product formation for the addition of arylboronic acids to 4-hydroxy-chromone.

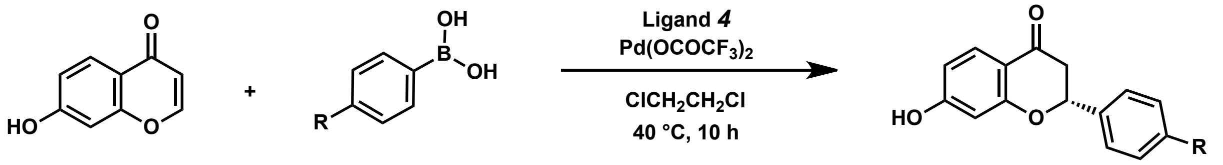

Entry	R =	*m/z* (Arylpalladium-cation)	*m/z* (Arylpalladium-chromone complex)	*m/z* (product) (Na^+)
1	4-Me-	401.08	563.11	277.08
2	4-Cl-	421.03	not observed	297.03
3	4-BnO-	493.11	655.14	369.11
4	4-I-	512.96	not observed	not observed

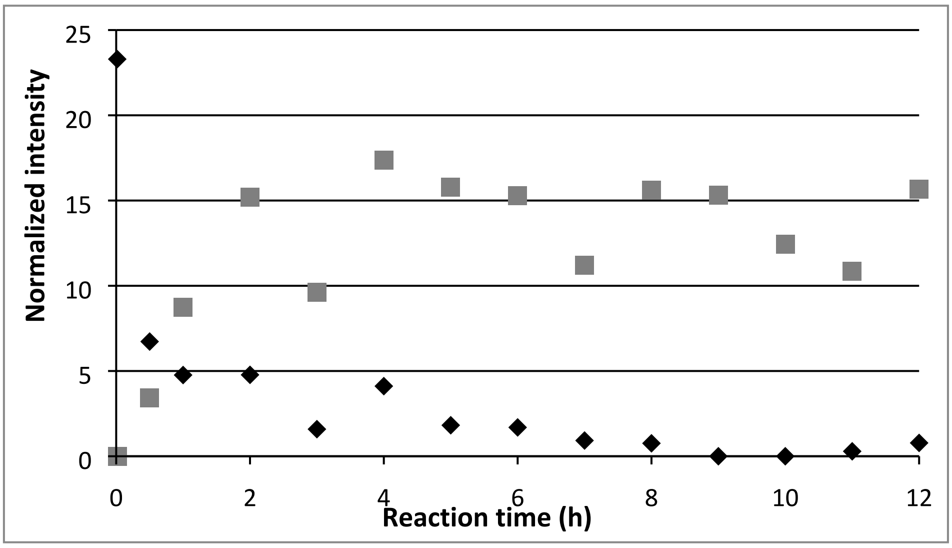

S8: Reaction monitoring in positive ion mode.

Table S3: Signal distribution between various states of the respective arylboronic acid. Intensities were normalized to the total ion count.

**Before reaction**	**Normalized Intensities**			
	**4-Me-**	**4-Bn-**	**4-I-**	**4-Cl-**
**Monomer**	0.00	0.00	0.02	0.00
**Dimer**	0.03	0.00	0.01	0.01
**Trimer**	4.38	0.12	1.83	2.11
**Tetramer**	2.37	0.39	22.51	9.23

**After reaction**	**Normalized intensities**			
	**4-Me-**	**4-Bn-**	**4-I-**	**4-Cl-**
**Monomer**	4.89	6.09	1.63	4.77
**Dimer**	0.01	0.00	0.00	0.08
**Trimer**	0.12	0.00	4.50	0.04
**Tetramer**	0.15	0.01	17.01	0.03

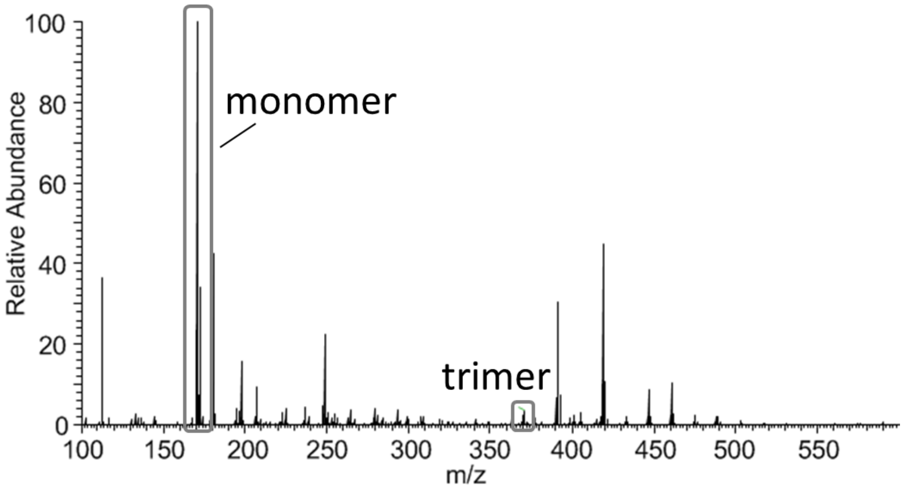

S9: 1 mmol **2** dissolved in CH_2Cl_2, spotted on a surface and sprayed with CH_2Cl_2.

Full reference 31 for Gaussian09:

Gaussian 09, Revision D.01, M. J. Frisch, G. W. Trucks, H. B. Schlegel, G. E. Scuseria, M. A. Robb, J. R. Cheeseman, G. Scalmani, V. Barone, B. Mennucci, G. A. Petersson, H. Nakatsuji, M. Caricato, X. Li, H. P. Hratchian, A. F. Izmaylov, J. Bloino, G. Zheng, J. L. Sonnenberg, M. Hada, M. Ehara, K. Toyota, R. Fukuda, J. Hasegawa, M. Ishida, T. Nakajima, Y. Honda, O. Kitao, H. Nakai, T. Vreven, J. A. Montgomery, Jr., J. E. Peralta, F. Ogliaro, M. Bearpark, J. J. Heyd, E. Brothers, K. N. Kudin, V. N. Staroverov, T. Keith, R. Kobayashi, J. Normand, K. Raghavachari, A. Rendell, J. C. Burant, S. S. Iyengar, J. Tomasi, M. Cossi, N. Rega, J. M. Millam, M. Klene, J. E. Knox, J. B. Cross, V. Bakken, C. Adamo, J. Jaramillo, R. Gomperts, R. E. Stratmann, O. Yazyev, A. J. Austin, R. Cammi, C. Pomelli, J. W. Ochterski, R. L. Martin, K. Morokuma, V. G. Zakrzewski, G. A. Voth, P. Salvador, J. J. Dannenberg, S. Dapprich, A. D. Daniels, O. Farkas, J. B. Foresman, J. V. Ortiz, J Cioslowski, and D. J. Fox, Gaussian, Inc., Wallingford CT, 2013.

Cartesian coordinates (Å), energies (hartree) and thermal corrections for optimized structures

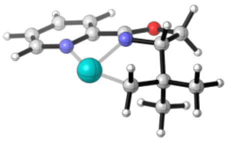

5

SCF Energy (M06, DCE):	-778.4972273
SCF Energy (M06, gas-phase):	-778.4131656
SCF Energy (M06L, gas-phase):	-778.739155
Enthalpy Correction:	0.269611
Free-Energy Correction:	0.212495

H	2.74259600	2.68615000	0.10808800
C	2.89980700	1.61262200	0.11825700
C	3.15612000	-1.11518500	0.11912800
C	1.82852900	0.75386500	-0.09141600
C	4.15979500	1.05579600	0.33223100
C	4.29031100	-0.32668900	0.32794400
H	5.02123000	1.69430100	0.49781300
H	5.25203300	-0.80155500	0.48776500
H	3.22495600	-2.19928400	0.11925500
N	1.94163400	-0.60170000	-0.08475800
C	0.46866200	1.22572900	-0.34734200
O	0.08750700	2.47749900	-0.14932200
C	-1.36826500	2.51843400	-0.40403900
H	-1.51531700	3.19568800	-1.24571300
H	-1.82742500	2.94150000	0.49175300
C	-1.76100900	1.06091100	-0.71169400
H	-2.23129300	0.98048900	-1.69936700
N	-0.44905700	0.40920900	-0.75928600
C	-2.58426200	0.22719500	0.29823400
C	-4.08043800	0.50903400	0.14137600
H	-4.42464700	0.31212100	-0.87832200
H	-4.31222600	1.55225800	0.38338000
H	-4.66526400	-0.11701100	0.82052800
C	-2.14772600	0.50630300	1.73611100
H	-1.06706600	0.37832400	1.87563100
H	-2.64432400	-0.18496400	2.42234600
H	-2.41632900	1.51772500	2.05645300
C	-2.29511400	-1.24026300	-0.07479500
H	-2.65812900	-1.50155200	-1.07690700
H	-2.68167300	-1.95093100	0.66775300
Pd	-0.26834100	-1.47179000	-0.14329400

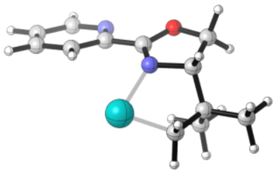

5-conf

SCF Energy (M06, DCE):	-778.486298
SCF Energy (M06, gas-phase):	-778.3891738
SCF Energy (M06L, gas-phase):	-778.7137197
Enthalpy Correction:	0.269222
Free-Energy Correction:	0.210047

H	1.73417900	-1.32135400	-1.11190600
C	2.39381700	-0.66724500	-0.53797500
C	4.01535500	1.12345700	0.72482400
C	1.98708400	0.62448300	-0.18207500
C	3.68954700	-1.05922400	-0.21949800
C	4.51581900	-0.14777800	0.43026100
H	4.05153600	-2.04667400	-0.48747100
H	5.53504100	-0.40796400	0.69587100
H	4.63915300	1.86219700	1.22241800
N	2.77589800	1.51549900	0.42949900
C	0.61654300	1.05770600	-0.46535700
O	0.32136800	2.34366400	-0.45585300
C	-1.10299200	2.47645000	-0.75726200
H	-1.17495400	2.99859900	-1.71234600
H	-1.53998700	3.09747500	0.02783400
C	-1.62416700	1.03688600	-0.80496400
H	-2.11787900	0.82160100	-1.76093300

N	-0.37408100	0.25179300	-0.73026400
C	-2.51746100	0.52529800	0.33976100
C	-3.95801900	1.02278300	0.17023100
H	-4.37086900	0.75361300	-0.80646800
H	-3.99998900	2.11277900	0.26908200
H	-4.60958400	0.60474000	0.94190500
C	-1.97604800	0.92918300	1.71027800
H	-0.93841800	0.60815300	1.85661100
H	-2.57378900	0.47249100	2.50358700
H	-2.02085900	2.01128000	1.86279300
C	-2.50364900	-0.99001200	0.18268900
H	-2.96203400	-1.34312900	-0.75069300
H	-2.88013100	-1.54199500	1.05414300
Pd	-0.59500100	-1.63900200	-0.03473300

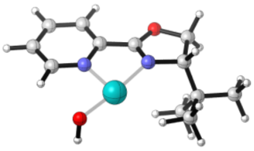

6

SCF Energy (M06, DCE):	-854.9268023
SCF Energy (M06, gas-phase):	-854.8380331
SCF Energy (M06L, gas-phase):	-855.1760095
Enthalpy Correction:	0.297231
Free-Energy Correction:	0.233187

H	2.04076900	3.33816300	0.08590400
C	2.38242200	2.30965000	0.13568100
C	3.13495100	-0.34848300	0.22782200
C	1.47084800	1.27999300	-0.03825000
C	3.71934500	1.98251500	0.36233700
C	4.09754200	0.64524000	0.40235100
H	4.45598500	2.76695300	0.50045500
H	5.12920400	0.35572900	0.56785400
H	3.35255200	-1.41265400	0.24509800
N	1.85381200	-0.02564600	0.02083700
C	0.04840600	1.43166200	-0.31356000
O	-0.56598800	2.60070500	-0.27833200
C	-1.98093200	2.31343800	-0.56596300
H	-2.23063800	2.85351200	-1.47971800
H	-2.55678900	2.72156800	0.26785500
C	-2.06714300	0.77774500	-0.70332600
H	-2.42159400	0.49742900	-1.70386400
N	-0.66058100	0.38363100	-0.58323900
C	-2.93642200	0.03303500	0.33699300
C	-4.38813600	0.47544100	0.14807700
H	-4.74556900	0.26719400	-0.86568400
H	-4.51685600	1.54626600	0.33911000
H	-5.04350200	-0.05305000	0.84532200
C	-2.46677100	0.33139200	1.75923000
H	-1.43197700	0.00390800	1.91997500
H	-3.09103200	-0.19801100	2.48376300
H	-2.52863900	1.39674500	2.00612900
C	-2.83279200	-1.46966500	0.06335700
H	-3.50735700	-2.03552800	0.71203000
H	-1.81837600	-1.85999700	0.28998700
H	-3.07154000	-1.72265700	-0.97455700
Pd	0.35206700	-1.37863800	-0.24541300
O	1.44259300	-2.95701000	0.04627400
H	1.33690200	-3.27544600	0.95412300

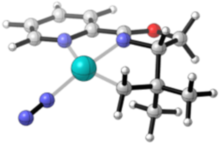

7

SCF Energy (M06, DCE):	-888.0090599
SCF Energy (M06, gas-phase):	-887.9384018
SCF Energy (M06L, gas-phase):	-888.2951845
Enthalpy Correction:	0.280653
Free-Energy Correction:	0.217055

H	-2.94306000	-2.85352400	0.29339900
C	-3.03150400	-1.77472600	0.21808100
C	-3.11447500	0.95810700	0.00896400
C	-1.91030300	-1.00492200	-0.06083500
C	-4.25190400	-1.12214700	0.38992600
C	-4.29468500	0.26160800	0.28022600
H	-5.15137100	-1.68919100	0.60596000
H	-5.22335300	0.80727700	0.40563300
H	-3.11082800	2.04149800	-0.07310100
N	-1.93997600	0.34995700	-0.16103500
C	-0.57719400	-1.56638200	-0.27859300
O	-0.26020200	-2.81487000	0.01803300
C	1.19250800	-2.94363400	-0.22627400
H	1.30896400	-3.69633500	-1.00626600
H	1.63121800	-3.30950600	0.70439400
C	1.64953400	-1.53582500	-0.65385700
H	2.11352000	-1.55904400	-1.64779200
N	0.37523800	-0.82288500	-0.74202700
C	2.52710900	-0.66862200	0.27878600
C	4.00313300	-1.05157500	0.14197100
H	4.35103200	-0.96127500	-0.89141100
H	4.17228100	-2.08374500	0.46870500
H	4.62998800	-0.40898700	0.76580700
C	2.08751500	-0.79665900	1.73679500
H	1.01715900	-0.59540800	1.86780300
H	2.62700700	-0.07689300	2.35818300
H	2.30180700	-1.78997600	2.14311800
C	2.33836500	0.77819800	-0.21706400
H	2.68088700	0.92301900	-1.24875700
H	2.81507700	1.51113200	0.44073100
Pd	0.29972000	1.12939100	-0.29401300
N	0.42371700	3.06105000	0.20525100
N	0.47895700	4.14151000	0.48140500

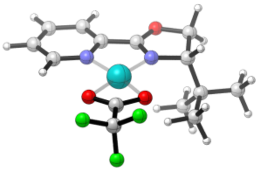

8

SCF Energy (M06, DCE): -1305.294542
SCF Energy (M06, gas-phase): -1305.211969
SCF Energy (M06L, gas-phase): -1305.561116
Enthalpy Correction: 0.31865
Free-Energy Correction: 0.242245

H	-4.23685600	2.35413000	-0.21319500
C	-3.20472400	2.62271600	-0.01467200
C	-0.54368700	3.18215800	0.45629900
C	-2.21524900	1.65784900	-0.11463300
C	-2.83057300	3.91824600	0.34236400
C	-1.48960200	4.19905900	0.57986700
H	-3.57982300	4.69772800	0.43146100
H	-1.16224100	5.19446000	0.85773300
H	0.51656600	3.34692500	0.62380200
N	-0.90664600	1.94084300	0.11951300
C	-2.40875000	0.25947800	-0.46149000
O	-3.59016600	-0.24825100	-0.74314500
C	-3.32803800	-1.64243700	-1.14301400
H	-3.50385000	-1.69211800	-2.21924000
H	-4.06024500	-2.25893600	-0.62052700
C	-1.86758800	-1.91381700	-0.73161700
H	-1.29651200	-2.34192900	-1.56382800
N	-1.38624500	-0.53849400	-0.49187300
C	-1.69727000	-2.82828100	0.50735400
C	-2.21898100	-4.21518200	0.12594200
H	-1.67336100	-4.62992600	-0.72809200
H	-3.28592000	-4.21288000	-0.12180500
H	-2.09066400	-4.90562700	0.96363300
C	-2.47495800	-2.27798500	1.70225400
H	-2.11491800	-1.28593400	1.99906700

H	-2.34674500	-2.93508900	2.56627400
H	-3.55207400	-2.21116800	1.51297400
C	-0.21498500	-2.92848000	0.86163800
H	-0.06633300	-3.66647400	1.65457400
H	0.18070800	-1.97613200	1.23217400
H	0.39387000	-3.23407800	0.00469700
Pd	0.39276900	0.38567500	-0.14371600
O	2.02085400	-0.92651800	-0.42102600
C	2.79445900	0.03452600	-0.13242300
O	2.30048900	1.17654500	0.13476900
C	4.30855100	-0.18921700	-0.05509100
F	4.96153900	0.95953800	-0.22656800
F	4.60466200	-0.68249100	1.15805500
F	4.69604600	-1.06443100	-0.98300900

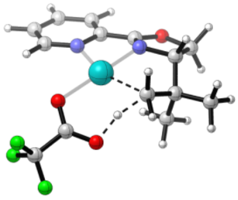

9TS

SCF Energy (M06, DCE):	-1305.253669
SCF Energy (M06, gas-phase):	-1305.170998
SCF Energy (M06L, gas-phase):	-1305.512582
Enthalpy Correction:	0.31206
Free-Energy Correction:	0.238506

H	-4.02803300	2.59157200	0.86877800
C	-3.00626800	2.76825200	0.54940300
C	-0.39015300	3.08473700	-0.25823600
C	-2.23191500	1.71043600	0.10045000
C	-2.43420000	4.04083500	0.57425800
C	-1.11633900	4.20021900	0.16411700
H	-3.01310400	4.89315000	0.91407500
H	-0.63911400	5.17373400	0.17014700
H	0.64836700	3.15452900	-0.56719900
N	-0.93601400	1.86808400	-0.29776600
C	-2.66707700	0.31898700	0.00308200
O	-3.74774600	-0.17997100	0.57015300
C	-3.66033000	-1.65123400	0.38946800
H	-4.56654300	-1.94934800	-0.13658800
H	-3.65810500	-2.08060100	1.39388900
C	-2.36859200	-1.88511900	-0.42194700
H	-2.59941000	-2.32972500	-1.39876900
N	-1.90644400	-0.51934600	-0.62719900
C	-1.17158300	-2.65966700	0.18528300
C	-1.42821100	-4.16288900	0.07058600
H	-1.54919100	-4.47441300	-0.97156900
H	-2.33166000	-4.45445800	0.61761400
H	-0.59729700	-4.73136800	0.49693300
C	-0.93531600	-2.27921700	1.64630600
H	-0.85603700	-1.19306900	1.78300600
H	0.00152000	-2.71506200	2.00488900
H	-1.72930700	-2.65218000	2.30058100
C	0.05893800	-2.28215300	-0.68067700
H	-0.15810000	-2.28770800	-1.75512400
H	0.83044600	-3.05741300	-0.56547800
Pd	0.01394000	-0.03058700	-0.63618900
O	1.96600700	0.64139800	-0.45136700
C	2.72180700	-0.23115200	0.06175300
O	2.39775300	-1.40763800	0.34900300
C	4.17044600	0.22355500	0.32824200
F	4.16238300	1.37458900	1.01579500
F	4.84983500	-0.68557700	1.01945900
F	4.78493500	0.42970100	-0.84524700
H	1.15179700	-1.59403100	-0.14062700

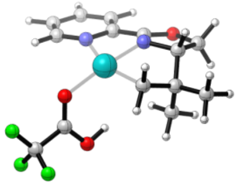

10

SCF Energy (M06, DCE): -1305.279383
SCF Energy (M06, gas-phase): -1305.201316
SCF Energy (M06L, gas-phase): -1305.540101
Enthalpy Correction: 0.317923
Free-Energy Correction: 0.241860

H	-4.32097400	2.12482300	0.97670600
C	-3.35503300	2.46826600	0.62119700
C	-0.88197100	3.20197700	-0.30715800
C	-2.43373800	1.55490800	0.12536700
C	-3.00194100	3.81699600	0.63812800
C	-1.75056700	4.18950900	0.16408300
H	-3.69615900	4.56095500	1.01483000
H	-1.43832000	5.22802000	0.15671100
H	0.10696500	3.45686400	-0.67873100
N	-1.20199600	1.90753100	-0.33055300
C	-2.69076900	0.11878000	0.03087100
O	-3.71089000	-0.48618100	0.62122400
C	-3.51822900	-1.93566700	0.40034200
H	-4.40414300	-2.28888500	-0.12717900
H	-3.47581300	-2.39583000	1.39032000
C	-2.22055000	-2.04761200	-0.42481600
H	-2.41702600	-2.50398000	-1.40352100
N	-1.87602900	-0.64185000	-0.62345500
C	-0.94428200	-2.69763600	0.16219500
C	-1.00195200	-4.22015300	0.02432800
H	-1.12604200	-4.52496400	-1.01901300
H	-1.83339200	-4.64008700	0.60164300
H	-0.08338900	-4.67931300	0.39991900
C	-0.75111100	-2.31834500	1.63063400
H	-0.80977100	-1.23226100	1.78792200
H	0.22786500	-2.66109100	1.98323700
H	-1.49344800	-2.78797500	2.28353100
C	0.21577000	-2.14158200	-0.69305900
H	0.13990600	-2.44720500	-1.74424500
H	1.19552700	-2.45222000	-0.30819900
Pd	0.02322100	-0.09387900	-0.75082400
O	2.09995400	0.36294100	-0.74556500
C	2.79197000	0.11769600	0.23725500
O	2.38208900	-0.42337600	1.35818500
C	4.29574200	0.44164200	0.26061600
F	4.66557900	0.99768300	-0.88490000
F	4.54707900	1.28664900	1.26729600
F	4.98734400	-0.68614700	0.45412700
H	1.41693400	-0.60161400	1.28266900

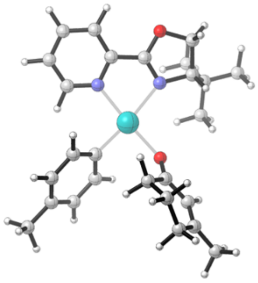

12

SCF Energy (M06, DCE): -1397.814532
SCF Energy (M06, gas-phase): -1397.738544
SCF Energy (M06L, gas-phase): -1398.334176
Enthalpy Correction: 0.573521
Free-Energy Correction: 0.475413

C	-3.03602500	0.16294900	-0.68719400
C	-4.05113500	-1.74882200	-1.17318900
C	-2.74194500	-2.02341200	-0.41124700
N	-2.13208700	-0.68517200	-0.33700200

H	-4.94048100	-2.18472800	-0.71446600
C	-2.83392000	1.60357200	-0.58021100
C	-3.83941200	2.52412300	-0.84710800
N	-1.59679400	1.97262300	-0.15211300
C	-3.57960300	3.87729000	-0.64912500
H	-4.80302900	2.16691100	-1.19363600
C	-1.35997200	3.27728400	0.04703300
C	-2.32425700	4.25410700	-0.18778400
H	-4.34640100	4.61953200	-0.84507200
H	-0.36564600	3.53053900	0.39966000
H	-2.07847300	5.29442100	-0.00595800
Pd	-0.23992900	0.38877800	0.11472700
C	2.74606900	-2.77489400	0.26579300
C	2.01393900	-1.62533300	-0.19541600
C	2.52663300	-0.86943200	-1.38128400
C	3.32457800	-1.75681200	-2.32533600
C	4.40728500	-2.51298100	-1.56970500
C	3.89122000	-3.19115900	-0.33835100
H	1.68398300	-0.36770300	-1.87119700
H	2.37159500	-3.27007200	1.15812400
H	2.65202300	-2.47831600	-2.80544600
H	3.76244100	-1.15766100	-3.12786900
H	4.89927700	-3.25070200	-2.21356900
H	5.21097700	-1.82638200	-1.25936400
H	3.15944000	-0.05995300	-0.98448100
O	0.98577800	-1.27673000	0.43675000
C	4.69290100	-4.31737000	0.20294600
H	4.29755100	-4.70326900	1.14352000
H	4.73558600	-5.13799800	-0.52260400
H	5.73253500	-4.00744800	0.35930200
C	1.34976300	1.54801200	0.40586000
C	2.14533400	1.36039100	1.53797100
C	1.73758600	2.48624500	-0.55494700
C	3.30321800	2.12111900	1.71128700
H	1.87494800	0.61972000	2.28767400
C	2.90413400	3.23245200	-0.37530400
H	1.14794900	2.63118600	-1.45954900
C	3.70397500	3.06760200	0.76125700
H	3.90893100	1.97116100	2.60334000
H	3.19951100	3.95143200	-1.13758300
O	-4.21060900	-0.29785100	-1.12051200
H	-2.07838300	-2.68089600	-0.98749500
H	-4.01583800	-2.02820400	-2.22920700
C	4.93539000	3.89583700	0.96700400
H	4.70895000	4.80886900	1.52876900
H	5.69673000	3.35517200	1.53501700
H	5.37750300	4.20722700	0.01724400
C	-2.93138500	-2.64887300	0.99504000
C	-3.85129900	-1.78246000	1.85504900
H	-3.98233000	-2.23721700	2.84096500
H	-3.43054100	-0.78312500	2.01253500
H	-4.85089800	-1.66966200	1.42062000
C	-1.57080100	-2.77635900	1.67617700
H	-0.86812200	-3.35659000	1.06761600
H	-1.11544900	-1.79831100	1.85976700
H	-1.67754500	-3.28366900	2.63984000
C	-3.53726000	-4.04125100	0.81272100
H	-2.89596800	-4.67967300	0.19493700
H	-3.65338500	-4.53237900	1.78306900
H	-4.52992900	-4.01244300	0.35033800

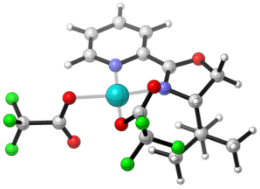

13

SCF Energy (M06, DCE): -1831.657114
SCF Energy (M06, gas-phase): -1831.617923
SCF Energy (M06L, gas-phase): -1831.969413
Enthalpy Correction: 0.353655
Free-Energy Correction: 0.257368

H	-0.29258400	5.00202600	-0.91055900
C	0.50541200	4.27314400	-0.81813500
C	2.43083200	2.31744500	-0.58398500
C	0.18876800	2.94080200	-0.59335500
C	1.84893300	4.62674300	-0.91746000
C	2.81979000	3.63756600	-0.79837200
H	2.13180900	5.66021200	-1.08903500
H	3.87566900	3.87266200	-0.87187200
H	3.14326900	1.50162400	-0.50505800
N	1.14168800	1.97767300	-0.47577300
C	-1.15639100	2.41149100	-0.43939400
O	-2.23404500	3.18809500	-0.51698700
C	-3.36541900	2.25318000	-0.44160600
H	-3.76183400	2.15853700	-1.45566200
H	-4.11227100	2.71292300	0.20741800
C	-2.76441300	0.94833600	0.10139600
H	-3.11289200	0.08182800	-0.47052100
N	-1.33681500	1.15527200	-0.19443100
C	-3.01239200	0.70374200	1.61405600
C	-4.50927100	0.44198600	1.79119800
H	-4.82857300	-0.43060200	1.21051300
H	-5.12701800	1.29588900	1.48940900
H	-4.73369100	0.23991800	2.84276700
C	-2.58511100	1.91648200	2.44182800
H	-1.51272600	2.11704200	2.33671000
H	-2.77107300	1.72757700	3.50309500
H	-3.13429100	2.82704600	2.17601200
C	-2.23418000	-0.52368400	2.08515700
H	-2.45952100	-0.72056200	3.13791400
H	-1.15069500	-0.39079300	1.99927100
H	-2.50408700	-1.41855800	1.51764500
Pd	0.40118100	0.06294800	-0.28611200
O	2.26247900	-0.77767900	-0.42901000
O	-0.41379500	-1.81115200	-0.18767500
C	2.75200900	-1.00183700	0.74737200
C	-1.35625100	-2.00297700	-1.04091100
O	-1.78346100	-1.25638700	-1.91461100
O	2.25753900	-0.77613700	1.84037000
C	-2.07795500	-3.35780300	-0.84760200
C	4.19149400	-1.55727700	0.64013300
F	-3.32986200	-3.11070500	-0.37438800
F	-2.21133700	-4.00627600	-2.01482600
F	-1.48086300	-4.18399100	0.02014000
F	4.27866000	-2.60988600	-0.18948300
F	5.00705100	-0.58372700	0.14762500
F	4.68048500	-1.92329800	1.82825800

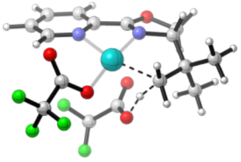

14TS

SCF Energy (M06, DCE): -1831.579275
SCF Energy (M06, gas-phase): -1831.545757
SCF Energy (M06L, gas-phase): -1831.892439
Enthalpy Correction: 0.346748
Free-Energy Correction: 0.254065

H	-0.66119100	0.28517400	1.85410800
C	-1.74009000	0.29194800	1.98278700
C	-4.45351600	0.20557100	2.21572600
C	-2.54216900	-0.61428300	1.28661100
C	-2.36247800	1.19933200	2.83146300
C	-3.74712000	1.16330800	2.94554400
H	-1.77401900	1.92407900	3.38525500
H	-4.27616100	1.86011400	3.58810300
H	-5.53797800	0.14617400	2.28618400
N	-3.87679200	-0.68007400	1.40553100
C	-1.93822600	-1.59209700	0.37189500
O	-2.55764900	-2.76742300	0.23988600
C	-1.77002800	-3.57496100	-0.67459700
H	-2.39483100	-3.78479300	-1.54487500
H	-1.53985700	-4.51452300	-0.16263000
C	-0.54360800	-2.71824900	-0.99334900
H	-0.46904800	-2.51307000	-2.07022100
N	-0.84671500	-1.45837900	-0.31037500
C	0.83505300	-3.18600000	-0.49479300
C	1.25808600	-4.42821000	-1.27937700
H	1.28533000	-4.23208000	-2.35654900
H	0.57109500	-5.26525400	-1.10531900
H	2.25504700	-4.76026600	-0.97424100
C	0.81675500	-3.49096100	1.00257700
H	0.50680000	-2.61802200	1.58799700
H	1.81792300	-3.77289700	1.34295900
H	0.14688400	-4.32271800	1.24693500
C	1.80578800	-2.03360700	-0.78243600
H	1.83039500	-1.77817800	-1.85076800
H	2.82797700	-2.37062600	-0.55928500
Pd	0.69224200	-0.14657500	-0.42293400
O	2.34088500	1.16233900	-0.37715100
C	3.22932300	0.68843400	0.36788600
O	3.24168500	-0.46997000	0.87599800
C	4.43507800	1.57782200	0.71192100
H	2.28833300	-1.09545200	0.23400200
F	5.57317900	0.97149600	0.33167200
F	4.37294200	2.76791800	0.11544800
F	4.48739100	1.77064500	2.04167900
O	-0.57077000	1.49990800	-0.44151900
C	-1.32129200	1.50489800	-1.49021100
O	-1.34836600	0.71873300	-2.42676500
C	-2.33775300	2.67193800	-1.43671200
F	-3.31353300	2.37894300	-0.53834400
F	-1.77396000	3.83116000	-1.05031600
F	-2.92873100	2.87827900	-2.61858400

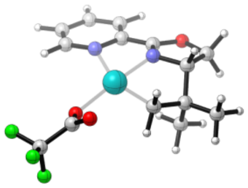

15

SCF Energy (M06, DCE):	-1304.856427
SCF Energy (M06, gas-phase):	-1304.823496
SCF Energy (M06L, gas-phase):	-1305.158222
Enthalpy Correction:	0.304547
Free-Energy Correction:	0.226685

H	-4.63162400	1.96801200	0.35265200
C	-3.62659900	2.36160200	0.23969500
C	-1.04334000	3.21933000	-0.06691900
C	-2.57727300	1.50296900	-0.07669000
C	-3.34307800	3.71477900	0.40590100
C	-2.03223000	4.15250000	0.25240100
H	-4.13508100	4.41413200	0.65544400
H	-1.76921700	5.19710800	0.38090300
H	-0.00429300	3.51664100	-0.18733000
N	-1.29469400	1.92132600	-0.23040100
C	-2.73761100	0.06394100	-0.25165600

O	-3.86509500	-0.59176100	0.05920600
C	-3.55306000	-2.02054200	-0.09905100
H	-4.25322600	-2.41675100	-0.83650100
H	-3.74054600	-2.49395200	0.86870400
C	-2.08180300	-2.06922500	-0.55564100
H	-1.99017600	-2.52876700	-1.54904700
N	-1.74902900	-0.65357400	-0.66024700
C	-0.98446300	-2.67432800	0.35512700
C	-0.98891800	-4.19930900	0.24151400
H	-0.83241000	-4.52331100	-0.79268700
H	-1.93563700	-4.62843000	0.59449400
H	-0.18835700	-4.63136600	0.84913200
C	-1.19599100	-2.26204800	1.81127300
H	-1.26800600	-1.17306900	1.91645000
H	-0.34341800	-2.58371400	2.41603300
H	-2.09252300	-2.71780000	2.24752600
C	0.35176800	-2.10458300	-0.17074600
H	0.60191200	-2.46742000	-1.17498500
H	1.18862100	-2.31931500	0.50108200
Pd	0.13342200	-0.08551500	-0.35686500
O	2.02413600	0.51595400	0.18338000
C	3.04964300	-0.08928000	-0.30544000
O	3.11390400	-1.04287900	-1.06713400
C	4.35734300	0.55043700	0.22486000
F	4.38687100	1.88569600	0.00482400
F	4.47423500	0.36539300	1.56196600
F	5.45142200	0.02884900	-0.34711300

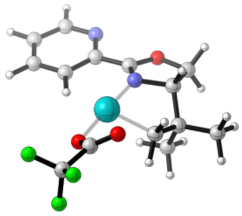

15-conf

SCF Energy (M06, DCE):	-1304.849362
SCF Energy (M06, gas-phase):	-1304.815566
SCF Energy (M06L, gas-phase):	-1305.148005
Enthalpy Correction:	0.304559
Free-Energy Correction:	0.225233

H	1.23880500	-1.79931600	0.51133100
C	2.28194100	-2.05069900	0.31627100
C	4.92777700	-2.52932800	-0.09920200
C	3.18279200	-1.05922100	-0.08660800
C	2.75817200	-3.34209300	0.50375100
C	4.10940700	-3.59174800	0.28683400
H	2.08656300	-4.13420000	0.82034200
H	4.52662000	-4.58478000	0.42069400
H	5.99159000	-2.68610200	-0.26851400
N	4.49094600	-1.28409100	-0.28379700
C	2.71714700	0.31363900	-0.31536800
O	3.63661900	1.28562400	-0.34235200
C	2.92889000	2.53963800	-0.52749100
H	3.32082300	3.00415500	-1.43448200
H	3.16553400	3.17817000	0.32963000
C	1.45334100	2.14465400	-0.62203300
H	1.03543000	2.39035400	-1.60792000
N	1.48822200	0.67766900	-0.49349700
C	0.46860200	2.65797700	0.44407600
C	0.22124300	4.15565900	0.24892600
H	-0.13384700	4.37567900	-0.76307300
H	1.13690600	4.73410600	0.42424300
H	-0.53263600	4.51872400	0.95342600
C	0.98506500	2.38846900	1.85593500
H	1.16047400	1.31966100	2.01987500
H	0.24603100	2.71483900	2.59320800
H	1.91402400	2.92924400	2.06867800
C	-0.82130100	1.88563900	0.18544600
H	-1.33522500	2.16630800	-0.74064100

H	-1.52441000	1.89330700	1.02457700
Pd	-0.39788500	-0.06793600	-0.16658300
O	-2.25837300	-0.78723800	0.27006500
C	-3.27783400	-0.28947700	-0.34440900
O	-3.33572800	0.63815600	-1.13716900
C	-4.57113400	-1.04278000	0.05279200
F	-4.47955500	-2.35797300	-0.24870000
F	-4.79927400	-0.95079200	1.38272600
F	-5.65149900	-0.56062100	-0.57462100

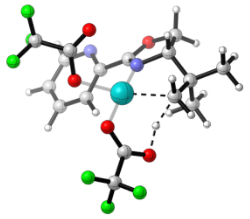

16TS

SCF Energy (M06, DCE):	-1831.586463
SCF Energy (M06, gas-phase):	-1831.554838
SCF Energy (M06L, gas-phase):	-1831.902445
Enthalpy Correction:	0.346881
Free-Energy Correction:	0.254617

H	-2.67252300	3.38832100	-1.56058600
C	-1.62622100	3.10346000	-1.59013800
C	0.98680900	2.24026800	-1.59676400
C	-1.27875700	1.76614700	-1.47599600
C	-0.60406100	4.04164200	-1.71428400
C	0.71368400	3.60333100	-1.72441400
H	-0.83744000	5.09843100	-1.79605400
H	1.53818900	4.30099000	-1.82212700
H	1.99607400	1.83993500	-1.60278700
N	0.01388800	1.33464100	-1.47204500
C	-2.23487300	0.68093600	-1.30376700
O	-3.54124700	0.83668800	-1.16136800
C	-4.02471700	-0.45824800	-0.64022400
H	-4.95497100	-0.67845100	-1.16350500
H	-4.20820500	-0.29729100	0.42426300
C	-2.88306200	-1.45495600	-0.92296600
H	-3.09508500	-2.01747500	-1.84414600
N	-1.79343000	-0.53067400	-1.24045000
C	-2.44154300	-2.48557400	0.16938000
C	-2.89188900	-3.88133800	-0.27833600
H	-2.45420000	-4.15603300	-1.24430600
H	-3.98328900	-3.93574000	-0.37261700
H	-2.58899700	-4.63633900	0.45322700
C	-3.06204100	-2.20397900	1.53804100
H	-2.85692600	-1.19372500	1.89343300
H	-2.65596300	-2.90587300	2.27271000
H	-4.14760600	-2.35460300	1.51431900
C	-0.88890100	-2.48794400	0.26925500
H	-0.45028600	-2.94196900	-0.63392900
H	-0.53104600	-3.14267700	1.07477000
Pd	0.09263600	-0.73680600	-0.70476200
O	1.97991000	-1.06117100	0.04481700
C	2.94443200	-0.67529500	-0.70966400
O	2.88963400	-0.11359900	-1.79913900
C	4.31865700	-0.97399100	-0.06368700
F	4.50063600	-0.20002200	1.02787700
F	4.41652300	-2.26029200	0.32678000
F	5.32880300	-0.72776000	-0.90910600
O	-0.34570100	-0.63026500	2.13738200
C	-0.89591000	0.51535700	2.01970700
O	-1.89631900	0.83903000	1.37025900
C	-0.11739200	1.66093900	2.70697000
F	0.65779400	2.25967100	1.75885600
F	-0.93831900	2.60508300	3.20005900
F	0.68862200	1.26018400	3.69592100
H	-0.56420700	-1.44396900	1.07608700

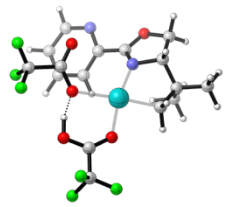

17

SCF Energy (M06, DCE):		-1831.629564
SCF Energy (M06, gas-phase):		-1831.605815
SCF Energy (M06L, gas-phase):		-1831.947309
Enthalpy Correction:		0.352205
Free-Energy Correction:		0.257112

H	-1.01261600	0.47424700	1.66441500
C	-1.88866400	1.10077500	1.51442100
C	-4.13604800	2.56574200	1.05139100
C	-2.91835800	0.68145600	0.67010500
C	-2.00986300	2.33978600	2.13020700
C	-3.14939100	3.09737700	1.88281200
H	-1.22221400	2.71100500	2.77924000
H	-3.27937500	4.07876600	2.32820600
H	-5.04873500	3.12427300	0.85151000
N	-4.04303500	1.37730400	0.45598700
C	-2.80407900	-0.59476700	-0.04626500
O	-3.93057500	-1.28618400	-0.25182900
C	-3.56793700	-2.51389100	-0.93473200
H	-4.04197200	-2.49294500	-1.91866400
H	-3.98239700	-3.34503800	-0.35572500
C	-2.04023200	-2.48690300	-0.98915700
H	-1.67638100	-2.56334200	-2.02234900
N	-1.71590700	-1.14541500	-0.47694500
C	-1.23221100	-3.47166000	-0.12580000
C	-1.34026600	-4.88480600	-0.69897100
H	-1.02404400	-4.91681600	-1.74717300
H	-2.36856900	-5.26356200	-0.64270300
H	-0.70746900	-5.57960800	-0.13848700
C	-1.71136100	-3.45737500	1.32483200
H	-1.61056600	-2.46210800	1.77143100
H	-1.11009700	-4.14711400	1.92456500
H	-2.75669000	-3.77248000	1.42056000
C	0.20506000	-2.95465800	-0.21495300
H	0.66869700	-3.20135200	-1.18146100
H	0.84694200	-3.31671600	0.59697200
Pd	0.28953700	-0.92276800	-0.20331800
O	2.39699600	-1.08303700	0.13278900
C	3.23943900	-0.19052800	0.25438000
O	3.08682400	1.08662100	0.18098800
C	4.70240600	-0.58790300	0.52390200
H	2.09801900	1.32709500	-0.02620000
F	4.82506300	-1.90554400	0.69207000
F	5.47421800	-0.21681700	-0.51163000
F	5.15705600	0.02639000	1.62763900
O	0.64002800	1.28727900	-0.29805000
C	-0.11992800	2.10207400	-0.95619800
O	-1.18557200	1.86935000	-1.49708000
C	0.42343100	3.55074700	-0.94760000
F	0.10540800	4.14882800	0.22797700
F	1.77189600	3.58538300	-1.05557800
F	-0.08389000	4.29562200	-1.93265200

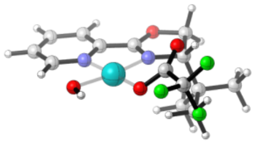

18

SCF Energy (M06, DCE):		-1381.296867
SCF Energy (M06, gas-phase):		-1381.264961
SCF Energy (M06L, gas-phase):		-1381.609667
Enthalpy Correction:		0.333503
Free-Energy Correction:		0.253549

H	4.69057600	1.07026600	-0.93411700
C	4.27777600	0.13789200	-0.56421700
C	3.09826100	-2.18048400	0.35608600
C	2.90660500	0.02918900	-0.36335700
C	5.07581900	-0.96670000	-0.28103200
C	4.47908300	-2.13684200	0.18355200
H	6.15040500	-0.91553200	-0.42503500
H	5.07126100	-3.01665600	0.40999200
H	2.55073300	-3.05631500	0.69931200
N	2.33510900	-1.11450600	0.09210100
C	1.93291800	1.07994500	-0.59576300
O	2.31116600	2.27500400	-1.05810600
C	1.06078900	2.99995200	-1.27831000
H	0.91337400	3.05956900	-2.36000700
H	1.20200800	4.00483000	-0.87558400
C	-0.01700700	2.17101800	-0.55933200
H	-0.88250400	1.98210100	-1.20192900
N	0.67735500	0.88720700	-0.35067500
C	-0.51267100	2.78482000	0.77622500
C	-1.18040700	4.12483800	0.45958500
H	-1.99672200	4.00077600	-0.26038400
H	-0.48000800	4.86444600	0.05545700
H	-1.60728000	4.55478400	1.37095200
C	0.65141300	2.99401800	1.74453200
H	1.12059200	2.04182400	2.01610000
H	0.29382100	3.45604100	2.66977200
H	1.42577400	3.65273300	1.33320800
C	-1.54934100	1.86039300	1.41013900
H	-1.91625500	2.29257500	2.34675400
H	-1.13703800	0.87206800	1.63623600
H	-2.40999600	1.72117500	0.74902700
Pd	0.29092100	-1.11055500	0.21997900
O	0.34952400	-3.00658800	0.72314400
O	-1.75856200	-1.27916700	0.32889500
C	-2.45631800	-0.67340800	-0.56357100
O	-2.09940600	0.05631400	-1.48004100
C	-3.96860300	-0.92302300	-0.33826800
F	-4.38372500	-0.27115500	0.77553200
F	-4.70961300	-0.47737100	-1.36195500
F	-4.25096500	-2.22946800	-0.16889600
H	-0.57128700	-3.28026800	0.83037400

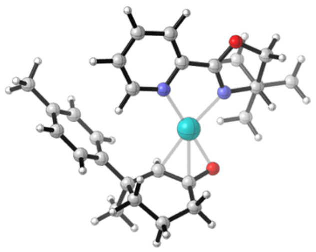

19

SCF Energy (M06, DCE):	-1397.820170
SCF Energy (M06, gas-phase):	-1397.741937
SCF Energy (M06L, gas-phase):	-1398.333687
Enthalpy Correction:	0.575088
Free-Energy Correction:	0.482819

C	-2.38359300	1.50425300	-0.76646600
C	-4.56419500	1.13861500	-0.94439800
C	-3.88808500	-0.08072400	-0.29026900
N	-2.46133800	0.26237400	-0.42836100
H	-5.37970300	1.56818300	-0.36078700
C	-1.09743000	2.17534100	-0.86692300
C	-0.96498700	3.51363100	-1.20884000
N	-0.03274400	1.36935700	-0.59845600
C	0.31342300	4.06228300	-1.28181700
H	-1.85620700	4.09782500	-1.41149300
C	1.19141700	1.90386100	-0.68856900
C	1.40226200	3.24157600	-1.02243000
H	0.45035900	5.10673500	-1.54206700
H	2.02211800	1.23796300	-0.49160600

H	2.42082100	3.61239300	-1.07250100
Pd	-0.56072500	-0.65623400	-0.16588500
C	1.14160800	-1.92280300	0.44455100
C	0.24364500	-2.75276200	-0.29229100
C	0.63873700	-3.52695000	-1.50970600
C	2.14954600	-3.60833300	-1.70052400
C	2.83963100	-2.29955600	-1.33868400
C	2.62521000	-1.89220900	0.12960400
H	0.17370000	-4.51591300	-1.43554800
H	0.85893200	-1.77018000	1.48884400
H	2.55907100	-4.42336300	-1.09404300
H	2.37311700	-3.87298600	-2.73738300
H	3.91256500	-2.39229000	-1.53535000
H	2.47099200	-1.49326400	-1.99157600
H	0.15312200	-3.04908900	-2.37029800
O	-1.00090700	-2.65889800	0.04719900
C	3.28603500	-2.92739200	1.07045200
H	2.85319300	-3.92549100	0.95692100
H	4.35830300	-2.98920400	0.85914500
H	3.16910800	-2.63310800	2.11707300
C	3.23796300	-0.53210100	0.45292800
C	2.90792400	0.12251500	1.64892400
C	4.15247400	0.09964000	-0.39488900
C	3.43316800	1.37040900	1.96033200
H	2.21660400	-0.34061700	2.35018500
C	4.68422600	1.35092800	-0.07785500
H	4.45141500	-0.36731000	-1.32819700
C	4.32938100	2.01670600	1.09730000
H	3.14495500	1.85464300	2.89108100
H	5.38916100	1.81757200	-0.76296600
O	-3.51173500	2.15655500	-1.01870400
H	-4.90283400	0.96108600	-1.96780800
H	-4.08320400	-0.99697200	-0.86050900
C	4.89378100	3.36115400	1.43784200
H	5.59782800	3.29586800	2.27380700
H	5.43158100	3.79984800	0.59398700
H	4.11093500	4.06227600	1.74487800
C	-4.29539300	-0.33104700	1.18450700
C	-4.05574000	0.91636100	2.03478800
H	-2.99730700	1.20113700	2.04369200
H	-4.64019600	1.77884800	1.69561900
H	-4.34594900	0.72422500	3.07139100
C	-3.48213000	-1.49480600	1.74643100
H	-3.56600800	-2.39113000	1.12382300
H	-2.41798400	-1.24822300	1.82278800
H	-3.83463300	-1.74861000	2.75045900
C	-5.77931500	-0.70277400	1.19764000
H	-5.97385100	-1.59664100	0.59527000
H	-6.10346600	-0.91861000	2.21945800
H	-6.42085000	0.10289300	0.82433000

$CF_3CO_2^-$

SCF Energy (M06, DCE):	-526.3054477
SCF Energy (M06, gas-phase):	-526.2290825
SCF Energy (M06L, gas-phase):	-526.2327498
Enthalpy Correction:	0.032651
Free-Energy Correction:	-0.00588

O	-1.59026500	1.14164600	0.00000800
C	-1.06431200	0.01267800	0.00000400
O	-1.53089500	-1.14388500	-0.00001300
C	0.50857800	0.01411700	0.00000000
F	1.02745900	-0.62988700	1.08881300
F	1.02745800	-0.62995700	-1.08877100
F	1.08993600	1.24397000	-0.00004100

**CF_3CO_2H**	SCF Energy (M06, DCE):		-526.7577417
	SCF Energy (M06, gas-phase):		-526.7508073
	SCF Energy (M06L, gas-phase):		-526.760328
	Enthalpy Correction:		0.046302
	Free-Energy Correction:		0.007851
O	1.50790700	-1.04953700	-0.00000300
C	0.93801300	0.15923400	0.00002600
O	1.50328800	1.22014700	-0.00000500
C	-0.59459100	0.00282500	-0.00000200
F	-0.99678100	-0.67616700	1.08881600
F	-1.18635800	1.19510100	-0.00012000
F	-0.99674700	-0.67636800	-1.08870400
H	2.46888100	-0.92033100	-0.00000600

**N_2**	SCF Energy (M06, DCE):		-109.4843991
	SCF Energy (M06, gas-phase):		-109.489865
	SCF Energy (M06L, gas-phase):		-109.5164513
	Enthalpy Correction:		0.008826
	Free-Energy Correction:		-0.012936
N	0.00000000	0.00000000	0.55485900
N	0.00000000	0.00000000	-0.55485900

**H_2O**	SCF Energy (M06, DCE):		-76.42614933
	SCF Energy (M06, gas-phase):		-76.4179516
	SCF Energy (M06L, gas-phase):		-76.41970383
	Enthalpy Correction:		0.025490
	Free-Energy Correction:		0.004072
O	0.00000000	0.00000000	0.11707800
H	0.00000000	0.76128400	-0.46831200
H	0.00000000	-0.76128400	-0.46831200
